# Cell Type-Specific Contributions of UBE3A to Angelman Syndrome Behavioral Phenotypes

**DOI:** 10.1523/ENEURO.0453-24.2025

**Published:** 2025-09-25

**Authors:** Nicholas W. Ringelberg, Renée E. Mayfield, Julia S. Lord, Graham H. Diering, Alain C. Burette, Benjamin D. Philpot

**Affiliations:** ^1^Neuroscience Curriculum, University of North Carolina, Chapel Hill, North Carolina 27599-7255; ^2^Neuroscience Center, University of North Carolina, Chapel Hill, North Carolina 27599-7255; ^3^Department of Cell Biology and Physiology, University of North Carolina, Chapel Hill, North Carolina 27599-7255; ^4^Carolina Institute for Developmental Disabilities, University of North Carolina, Chapel Hill, North Carolina 27599-7255

**Keywords:** Angelman, behavior, mouse, neurodevelopment, sleep, ube3a

## Abstract

Angelman syndrome (AS) is a rare neurodevelopmental disorder caused by loss of expression of the maternal *UBE3A* allele and is characterized by a constellation of impactful neurologic symptoms. While previous work has uncovered outsized contributions of GABAergic neuron-selective *Ube3a* deletion to seizure susceptibility and electroencephalography (EEG) phenotypes in a mouse model of AS, the neuronal populations governing a broader range of behaviors have not been studied. Here, we used male and female mice to test the consequences of *Ube3a* deletion from GABAergic or glutamatergic neurons across a well-characterized battery of AS-relevant behaviors. Surprisingly, we observed deficits in numerous motor and innate behaviors in mice with glutamatergic *Ube3a* deletion and relatively few consequences of GABAergic *Ube3a* deletion. Furthermore, genetic *Ube3a* reinstatement in glutamatergic neurons rescued multiple motor and innate behaviors. When tested for sleep–wake behaviors, the selective loss of *Ube3a* from glutamatergic neurons disrupted sleep similarly to that of AS model mice (*Ube3a^m–/p+^*), and glutamatergic *Ube3a* reinstatement overcame the lack of active cycle “siesta” and decreased REM phenotypes observed in AS model mice. Altogether, this work demonstrates a major role of glutamatergic neuron UBE3A loss in mediating multiple AS behavioral features, suggesting a divergence from the circuitry underlying enhanced seizure susceptibility. Our findings imply that neuronal cell type-agnostic UBE3A reinstatement is likely required for successful AS genetic therapies—with reinstatement of UBE3A in GABAergic neurons necessary for overcoming epileptic and EEG phenotypes, and reinstatement in glutamatergic neurons necessary for overcoming most other behavioral phenotypes.

## Significance Statement

Angelman syndrome (AS), a severe neurodevelopmental disorder caused by loss of neuronal UBE3A, is characterized by symptoms such as motor impairment, lack of speech, seizures, and disrupted sleep. While clinical trials aim to restore UBE3A in AS individuals, the neuronal populations responsible for key symptoms remain unclear. Using an AS mouse model, we identify a key role for excitatory neuron loss of UBE3A in motor, innate behavior, and sleep phenotypes, distinct from the previously described impact of inhibitory neuron loss of UBE3A on seizure and electroencephalography phenotypes. These data improve our understanding of the mechanisms by which UBE3A loss leads to symptoms, potentially guiding future therapies.

## Introduction

Many core symptoms of autism spectrum disorder (ASD) and other neurodevelopmental disorders are posited to arise from disruptions in the interplay between excitatory and inhibitory neurons, deemed an “excitatory–inhibitory (E–I) imbalance” (Rubenstein and Merzenich, 2003). While this hypothesis can oversimplify complex network deficits, it has provided a useful framework to study how neuronal populations interact in the neurotypical brain and animal models of disease (Nelson and Valakh, 2015; Sohal and Rubenstein, 2019). One strategy to understand how this interplay between excitatory and inhibitory neuronal populations causes symptoms pertinent to neurodevelopmental disorders is to selectively perturb the expression of key risk genes in these broad neuronal populations and then study the resulting effects on behavior. Indeed, selective deletion of neurodevelopmental disorder-linked genes in excitatory versus inhibitory neurons has revealed their divergent contributions to behavioral phenotypes in multiple mouse models of neurodevelopmental disorders, providing insights regarding the mechanisms underlying these symptoms (Chao et al., 2010; Meng et al., 2016; Kim et al., 2024).

Angelman syndrome (AS), a severe neurodevelopmental disorder caused by loss of the maternal allele of E3 ubiquitin ligase *UBE3A*, is characterized by a constellation of symptoms including recurrent seizures (>90% of individuals), motor impairment, sleep disruption, intellectual disability, and absence of speech (Williams et al., 2010; Thibert et al., 2013; Buiting et al., 2016). Interestingly, mice with *Ube3a* deletion restricted to GABAergic neurons display seizure susceptibility that is much more severe than that of mice with pan-neuronal loss of *Ube3a*, suggesting a key role of GABAergic UBE3A loss in seizures (Judson et al., 2016; Gu et al., 2019). GABAergic *Ube3a* deletion also mediates increased cortical electroencephalography (EEG) power in the delta frequency (2–4 Hz), a phenotype characteristic of AS individuals and model mice (Judson et al., 2016; Sidorov et al., 2017). This finding has particular clinical relevance, as EEG delta power robustly correlates with the severity of several AS symptoms, implicating this phenotype as a potential biomarker for measuring the efficacy of novel treatments (Hipp et al., 2021; Ostrowski et al., 2021; Spencer et al., 2022).

While deletion of *Ube3a* from GABAergic neurons plays a key role in seizure and EEG phenotypes of AS model mice, the neuronal populations responsible for a broader range of behaviors have not been assessed. Here, we investigated the contributions of GABAergic and glutamatergic neuron UBE3A loss to AS-related behaviors using a well-characterized behavioral battery (Sonzogni et al., 2018; Judson et al., 2021; Syding et al., 2022). Surprisingly, deletion of *Ube3a* from GABAergic neurons yielded few behavioral deficits, while *Ube3a* deletion from glutamatergic neurons drove AS-like behaviors in multiple motor and innate behavioral tasks. Accordingly, genetic reinstatement of *Ube3a* in glutamatergic neurons improved performance in many behavioral tasks, further suggesting UBE3A expression in this neuronal population plays a key role. To test other clinically relevant phenotypes, we used a noninvasive monitoring system to study sleep–wake behavior in mouse models with cell class-specific conditional *Ube3a* manipulation. These studies yielded similar results, predominantly implicating glutamatergic deletion of *Ube3a* in the altered sleep–wake behavior of AS mice, but revealing a role of GABAergic *Ube3a* deletion in a sleep fragmentation phenotype. Taken together, our data support a predominant role of glutamatergic deletion of *Ube3a* in many of the motor and innate behaviors of AS model mice, contrasting the circuitry mediating seizure and EEG phenotypes. As clinical trials are currently underway to reinstate UBE3A in AS individuals, our findings suggest that gene delivery strategies must effectively target both excitatory and inhibitory neurons, in a balanced manner, for optimal symptom improvement.

## Materials and Methods

### Animals

All procedures were approved by the Institutional Animal Care and Use Committee of the University of North Carolina at Chapel Hill and were performed in accordance with the guidelines of the US National Institutes of Health. Mice were raised on a 12 h light/dark cycle (lights on at 7 A.M.) and were housed in groups of 2–5 mice per cage with *ad libitum* access to food (PicoLab 5V5M chow) and water. Mice of both sexes were used in approximately equal genotypic ratios. AS model mice do not display sexually dimorphic phenotypes in the battery of behavioral tests used (Sonzogni et al., 2018)—open field, marble burying, rotarod, and nest building—so data from male and female mice are shown combined for these tests. All experiments and post hoc data collection were performed by experimenters blinded to genotype.

All mice were maintained on a C57BL/6J background. Mice with neuron type-specific deletion of *Ube3a* were generated by crossing male mice heterozygous for Gad2-IRES-Cre (JAX 028867; Taniguchi et al., 2011) or Vglut2-IRES-Cre (JAX 028863; Vong et al., 2011) to female mice heterozygous (paternal inheritance) or homozygous for the *Ube3a-Flox* allele (Judson et al., 2016). To generate mice with glutamatergic neuron type-specific reinstatement of *Ube3a*, male mice heterozygous for Vglut2-IRES-Cre were crossed to females heterozygous (paternal inheritance) for the *Ube3a lox-STOP-lox* construct (Silva-Santos et al., 2015). Maternal *Ube3a-*deficient mice (*Ube3a^m−/p+^* = AS model mice) were produced by crossing female paternal *Ube3a-*deficient mice (JAX 016590) to congenic C57BL/6J males. The experimental models used in each figure are summarized in Table 1.

**Table 1.** Summary of experimental models used, nomenclature, and controls used for each figure

Figure	Genetic manipulation and nomenclature	Experimental groups (*n*)
1, 1-1*A–E*	GABAergic *Ube3a* Deletion (Gad2-Cre::*Ube3a^mFLOX/p+^*)	WT (2) Flox (20) Cre (2) Cre::Flox (15–19)
1-1*F*	GABAergic *Ube3a* Deletion (Gad2-Cre::*Ube3a^mFLOX/p+^*)	WT (5) Flox (2) Cre (8) Cre::Flox (10)
2, 2-1	Glutamatergic *Ube3a* Deletion (Vglut2-Cre::*Ube3a^mFLOX/p+^*)	WT (14) Flox (8) Cre (10–11) Cre::Flox (14)
3, 3-2	Glutamatergic *Ube3a* Reinstatement (Vglut2-Cre::*Ube3a^mSTOP/p+^*)	WT (13) Cre (16) mSTOP (14–15) Cre::mSTOP (14)
4, 8, 4-1, 8-1	Pan-neuronal *Ube3a* Deletion (*Ube3a^m−/p+^* or AS)	WT (19) AS (21)
5, 5-1	GABAergic *Ube3a* Deletion (Gad2-Cre::*Ube3a^mFLOX/p+^*)	WT (10) Flox (8) Cre (12) Cre::Flox (12)
6, 6-1	Glutamatergic *Ube3a* Deletion (Vglut2-Cre::*Ube3a^mFLOX/p+^*)	WT (11) Flox (13) Cre (17) Cre::Flox (15)
7, 9, 7-1, 9-1, 9-2	Glutamatergic *Ube3a* Reinstatement (Vglut2-Cre::*Ube3a^mSTOP/p+^*)	WT (10) Cre (11) mSTOP (15) Cre::mSTOP (15)

We genotyped mice using the following polymerase chain reaction (PCR) primers:

*Ube3a*: Ube3a P1 (5′-ACT TCT CAA GGT AAG CTG AGC TTG C-3′), Ube3a P2 (5′-GCT CAA GGT TGT ATG CCT TGG TGC T-3′), Ube3a P3 (5′-TGC ATC GCA TTG TGT GAG TAG GTG TC-3′)

*Ube3a^FLOX^*: Komp 1.2F (5′-AAA ATT GGG TAT GCG AGC TG-3′), Komp 1.5R (5′-GGG GTC TAA GGG CCT ATG AA-3′)

*Ube3a^STOP^*: LSL F (5′-GTA CAT TGC ATT TGC CGT GA-3′), LSL R (5′-GGG GAA CTT CCT GAC TAG GG-3′)

Cre: All Cre F (5′-GAT GGA CAT GTT CAG GGA TCG CC-3′), All Cre R (5′-CTC CCA CCG TCA GTA CGT GAG AT-3′).

### Behavioral testing

Mice 2–4 months of age were tested in a well-characterized battery of behavioral tests. Behaviors were performed in the following order with at least 48 h between experiments. Week 1, open field, marble burying; Week 2, rotarod; Week 3, nest building. All behavioral testing was conducted during the light cycle at a similar time of day within each cohort. Mice were allowed to acclimate to the behavioral testing room for at least 30 min prior to starting each experiment.

Unless otherwise stated, mice were run through all tests in the behavioral battery. Of note, however, Gad2-Cre::*Ube3a^mFLOX/p+^* mice sometimes show premature mortality in adulthood, presumably from spontaneous seizures (Judson et al., 2016), and spontaneous mortality was observed in Vglut2-Cre::*Ube3a^mSTOP/p+^* mice as well. Therefore, there was some drop-off in sample size for these groups over the period of the behavioral battery.

Separate cohorts of mice were evaluated for sleep phenotypes (described below), except for a small subset of Vglut2-Cre::*Ube3a^mSTOP/p+^* mice and littermate controls that were tested in sleep chambers after completing the behavioral battery.

#### Open field

Mice were placed in a novel, sound-attenuated arena (40 cm × 40 cm, ∼650 lux) for 30 min and were video recorded. Arenas were cleaned with ethanol and water and then dried thoroughly between mice. Time in center was defined as the amount of time the animal's center point resided in the central 20 cm × 20 cm of the arena. Total distance traveled and time in center were quantified using EthoVision XT 15.0 software.

#### Marble burying

Mice were tested in a clean cage placed within the open field arena with the same lighting conditions. Cages were filled with 3 L of 1/8-inch-diameter irradiated corncob bedding (Anderson Lab), on top of which 20 black marbles were placed in an equidistant 5 × 4 grid. Mice were placed in cages with marbles for 30 min with a clear, ventilated plexiglass lid. Before and after images of each cage were taken and used to objectively compute the percentage of marble area obscured by bedding using ImageJ software. Manual counts of the number of marbles at least two-thirds buried were also performed using these images and are reported in the Extended Data.

#### Accelerating rotarod

Mice were placed on the accelerating rotarod (Ugo Basile Model 47650) at an initial speed of 3 revolutions per minute (rpm), steadily increasing to 30 rpm over 5 min. Mice underwent three consecutive trials on the first day, and two additional trials 48 h later, with an intertrial interval of ∼1 min. Latency to the animal's first passive rotation and latency to 3× rotations or fall were recorded.

#### Nest building

**Figure 1.**
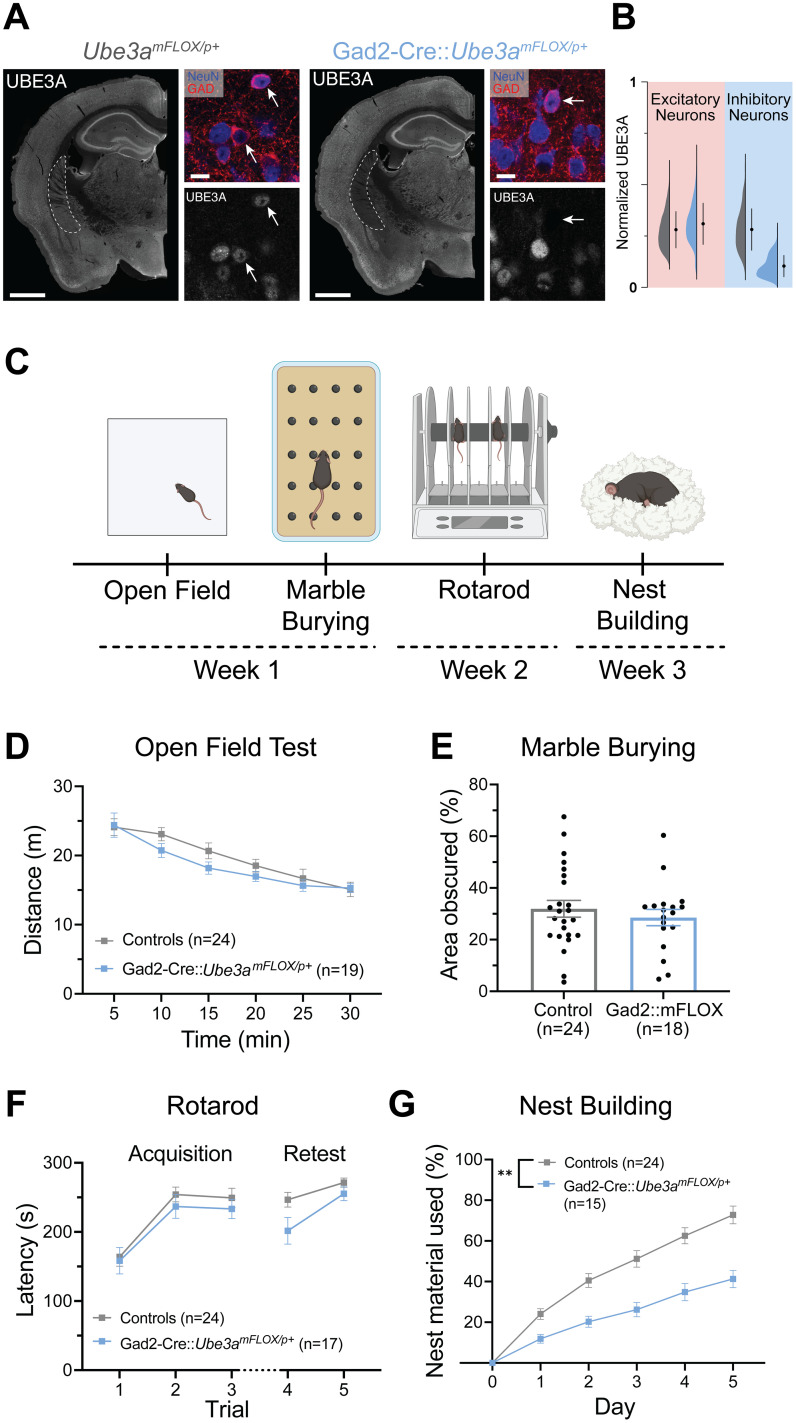
Mice with maternal allele *Ube3a* deletion from GABAergic neurons (Gad2-Cre::*Ube3a^mFLOX/p+^*) exhibit only modest behavioral deficits. ***A***, UBE3A immunostaining in *Ube3a^mFLOX/p+^* and Gad2-Cre::*Ube3a^mFLOX/p+^* mice. Dashed lines indicate the largely GABAergic caudate putamen, in which UBE3A is robustly deleted in Gad2-Cre::*Ube3a^mFLOX/p+^* mice. Zoom images demonstrate cell type-specific UBE3A expression in the somatosensory cortex. Arrows indicate NeuN+, GAD*^+^* neurons. Hemi-section scale bar, 1 mm. Zoom image scale bar, 10 µm. ***B***, Nuclear UBE3A intensity of individual excitatory (NeuN+, GAD−) and inhibitory (NeuN+, GAD+) cells in the somatosensory cortex displayed as violin plots with mean ± SD. ***C***, Schematic of behavioral battery. ***D***, Distance traveled in the open field across 5 min bins. Two-way RM ANOVA. ***E***, Quantification of marble burying behavior by threshold-based analysis of area obscured. Unpaired *t* test. ***F***, Latency to fall or first passive rotation on the rotarod across each acquisition (Day 1) and retest (Day 2) trial. Two-way RM ANOVA. ***G***, Quantification of percent nesting material used across 5 d test. Two-way RM ANOVA. Behavioral data presented as mean ± SEM. *p < 0.05, **p < 0.01. Individual data points labeled by genotype and sex are reported in Extended Data Figure 1-1. Statistical tests and output statistics for all figures are reported in Extended Data Figure 1-2. Created with BioRender (https://BioRender.com/a9rsfhy).

**Figure 2.**
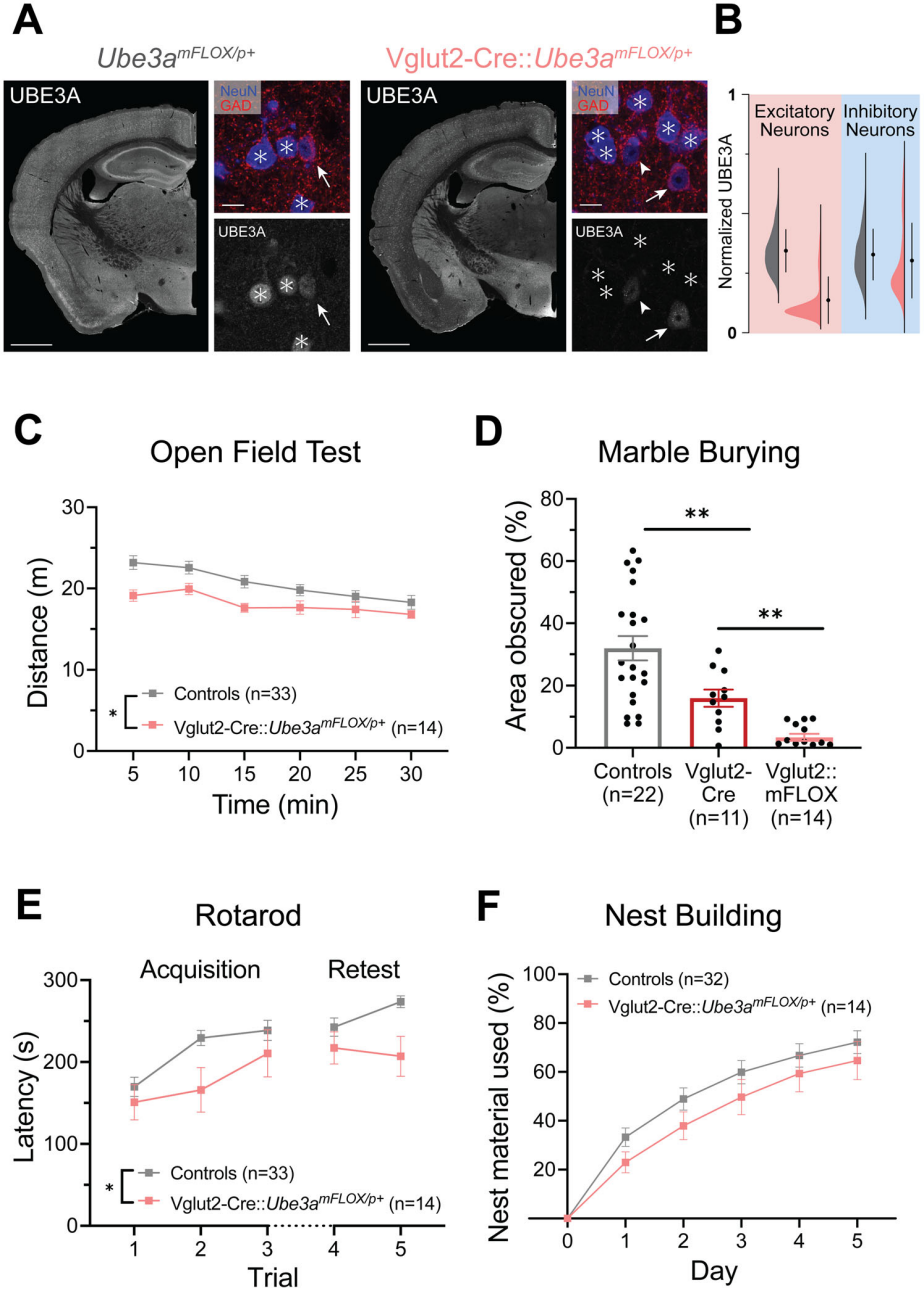
Mice with *Ube3a* deletion from glutamatergic neurons (Vglut2-Cre::*Ube3a^mFLOX/p+^*) exhibit motor and innate behavioral deficits. ***A***, UBE3A immunostaining in *Ube3a^mFLOX/p+^* and Vglut2-Cre::*Ube3a^mFLOX/p+^* mice. Zoom images demonstrate cell type-specific UBE3A expression in the somatosensory cortex. Asterisks indicate excitatory (NeuN+, GAD−) cells. Arrows indicate inhibitory (NeuN+, GAD+) neurons. Arrowhead indicates example excitatory neuron with intact UBE3A expression in Vglut2-Cre::*Ube3a^mFLOX/p+^* mouse. Hemi-section scale bar, 1 mm. Zoom image scale bar, 10 µm. ***B***, Nuclear UBE3A intensity of individual excitatory (NeuN+, GAD−) and inhibitory (NeuN+, GAD+) cells in the somatosensory cortex displayed as violin plots with mean ± SD. ***C***, Distance traveled in the open field across 5 min bins. Two-way RM ANOVA. ***D***, Quantification of marble burying behavior by threshold-based analysis of area obscured in Cre-negative controls, Vglut2-Cre controls, and Vglut2-Cre::*Ube3a^mFLOX/p+^* mice. Brown–Forsythe test with Dunnett's T3 post hoc multiple comparisons. ***E***, Latency to fall or first passive rotation on the rotarod across each acquisition (Day 1) and retest (Day 2) trial. Two-way RM ANOVA. ***F***, Quantification of percent nesting material used across 5 d test. Two-way RM ANOVA. Behavioral data presented as mean ± SEM. *p < 0.05, **p < 0.01. Individual data points labeled by genotype and sex are reported in Extended Data Figure 2-1.

**Figure 3.**
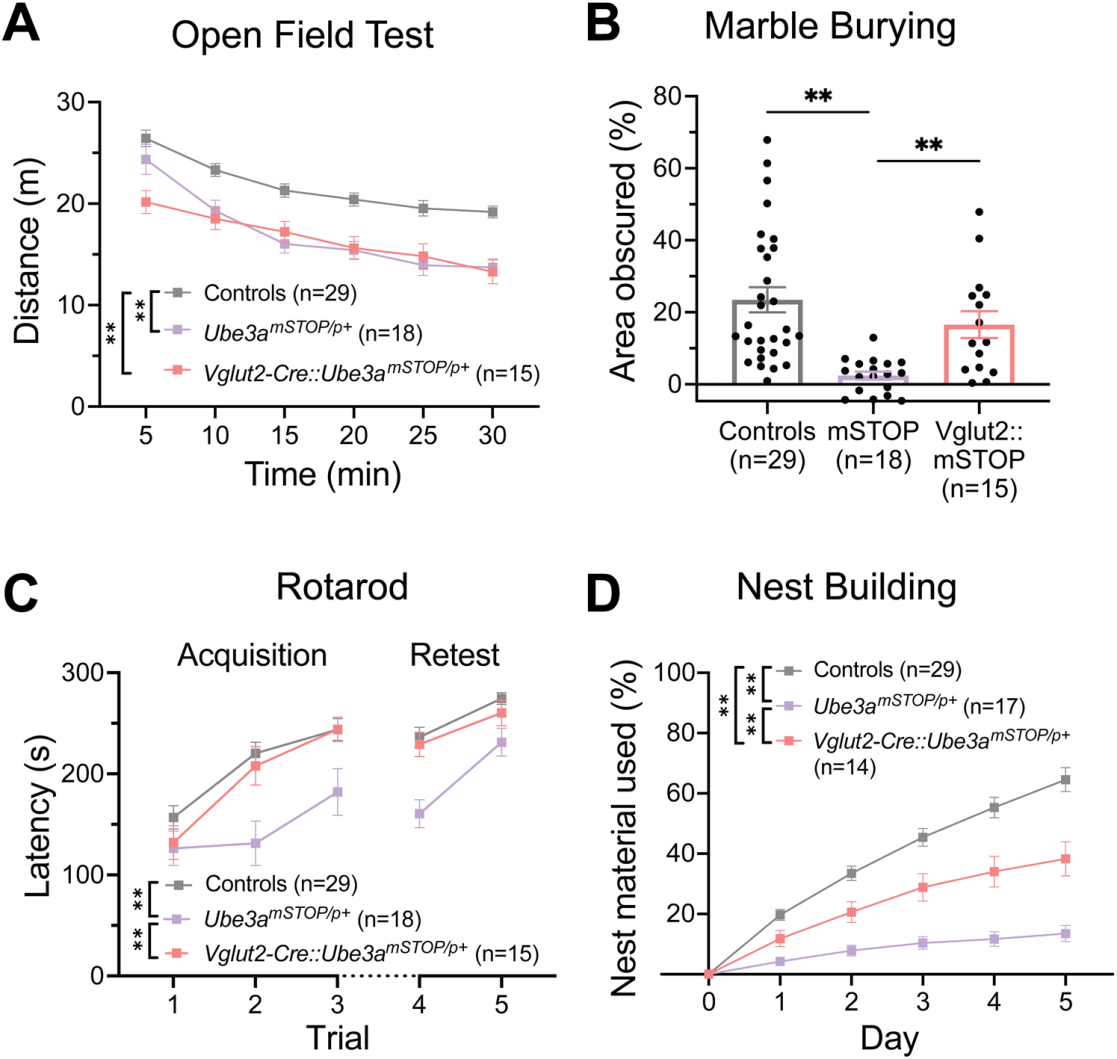
Glutamatergic neuron-selective *Ube3a* reinstatement (Vglut2-Cre::*Ube3a^mSTOP/p+^*) rescues AS motor and innate behavioral deficits. ***A***, Distance traveled in the open field across 5 min bins. Two-way RM ANOVA with Tukey's post hoc comparisons of genotype effect. ***B***, Quantification of marble burying behavior by threshold-based analysis of area obscured. Brown–Forsythe test with Dunnett's T3 post hoc multiple comparisons. ***C***, Latency to fall or first passive rotation on the rotarod across each acquisition (Day 1) and retest (Day 2) trial. Two-way RM ANOVA with Tukey's post hoc comparisons of genotype effect. ***D***, Quantification of percent nesting material used across 5 d test. Two-way RM ANOVA with Tukey's post hoc comparisons of genotype effect. Data presented as means ± SEM. *p < 0.05, **p < 0.01. Histological validation of glutamatergic UBE3A reinstatement in the Vglut2-Cre::*Ube3a^mSTOP/p+^* model is reported in Extended Data Figure 3-1. Individual data points labeled by genotype and sex are reported in Extended Data Figure 3-2.

Mice were single-housed for 3–5 d before beginning the nest building experiment. On Day 1, each animal's facility-provided nesting material was removed and replaced with 11 ± 1 g of extra-thick blot filter paper (Bio-Rad 1703966) cut into eight evenly sized rectangles. For 5 consecutive days, the amount of unused blot paper was weighed, recorded, and placed back in the cage. Across all experimental groups, three mice were excluded from this task because they showed noticeable weight loss and “ruffled” fur upon single-housing, suggestive of stress (Fig. 1*G*, one Gad2-Cre::*Ube3a^mFLOX/p+^* mouse; Fig. 2*F*, one Vglut2-Cre control mouse; Fig. 3*D*, one *Ube3a^mSTOP/p+^* mouse).

#### Sleep recordings

Mice were single-housed in 15.5 cm^2^ chambers with food, water, and nesting material in a dedicated sleep behavior monitoring room. Mice were given two full dark cycles to acclimate to single housing, after which sleep data were recorded for the following 7–9 d. Cage bedding was changed once during the experiment, and data from 7 A.M. of this day to 7 A.M. of the following day were excluded from analysis.

Sleep–wake behavior was quantified using a noninvasive monitoring system, PiezoSleep 2.0 (Signal Solutions), as previously described (Martin et al., 2024). Briefly, the PiezoSleep system uses a piezoelectric mat underneath the floor of each cage to measure pressure changes due to movements of the mouse. Signals were analyzed using custom software (SleepStats, Signal Solutions) to extract movement and respiratory patterns from pressure data, which was used to determine sleep versus wake states. Sleep was characterized by respiratory patterns typical of a sleeping mouse, and wake was characterized by volitional movements and the absence of sleep-typical signals. Average sleep bout duration was also extracted: sleep bout onsets were identified by 30 s periods composed of >50% sleep and terminations by 30 s periods of <50% sleep. This approach has been validated using EEG, EMG, and visual observation (Donohue et al., 2008; Mang et al., 2014).

Estimates of REM and NREM sleep were extracted using SleepStats version 4.0 (Signal Solutions) and were determined by periods of irregular breathing characteristic sleep state transitions (Yaghouby et al., 2016; Vanneau et al., 2021).

Average percent time sleeping and estimates of REM and NREM were calculated in 1 h bins, and average sleep bout durations were calculated using 4 h bins. One cohort, in which control mice did not demonstrate a clear “siesta” during the dark cycle, was excluded from analysis. This was attributed to a recent transfer of the mouse colony to a new vivarium, as consistent siesta behavior in C57BL/6J mice is a good prognosticator of appropriately quiet and relatively undisturbed animal care conditions.

### Immunohistochemistry

Free-floating sections were rinsed twice in PBS (5 min each), followed by PBS containing 0.1% Triton X-100 (PBS-T). Sections were blocked for 30 min at room temperature in PBS-T containing 10% fetal bovine serum and then incubated overnight at room temperature with primary antibodies (GAD, 1:1,000; UBE3A, 1:1,000; NeuN, 1:1,000). After PBS-T rinses, sections were incubated with fluorescence-conjugated secondary antibodies and counterstained with DAPI. Mounted sections were air-dried and coverslipped using Vectashield Plus (Vector Laboratories, H-1900). Images were acquired using a Leica STELLARIS 8 FALCON microscope and quantified using QuPath software (RRID: SCR_018257) and InstanSeg (https://github.com/instanseg).

For UBE3A antigen detection, we used the mouse monoclonal antibody 3E5 (Sigma-Aldrich, catalog #SAB1404508, RRID:AB_10740376). For NeuN antigen detection, we used a guinea pig polyclonal antibody (Millipore, catalog #ABN90, RRID:AB_11205592) generated against a GST-tagged recombinant fragment corresponding to the first 97 amino acids of mouse NeuN. For GAD antigen detection, we used a rabbit monoclonal antibody (Abcam, catalog #ab183999, RRID:AB_3662875) that recognizes both GAD65 and GAD67 isoforms. The immunogen used to produce this antibody is proprietary.

For quantification of cell type-specific UBE3A expression levels in conditional knock-out and reinstatement models, nuclear UBE3A intensity was extracted in individual cells in the somatosensory cortex. For each experimental condition, quantification was performed using three animals per condition, three sections per animal, and both hemispheres of each section. Excitatory neurons were defined as NeuN+, GAD^−^ cells, and inhibitory neurons were defined as NeuN+, GAD*^+^* cells.

### Experimental design and statistical analysis

**Figure 4.**
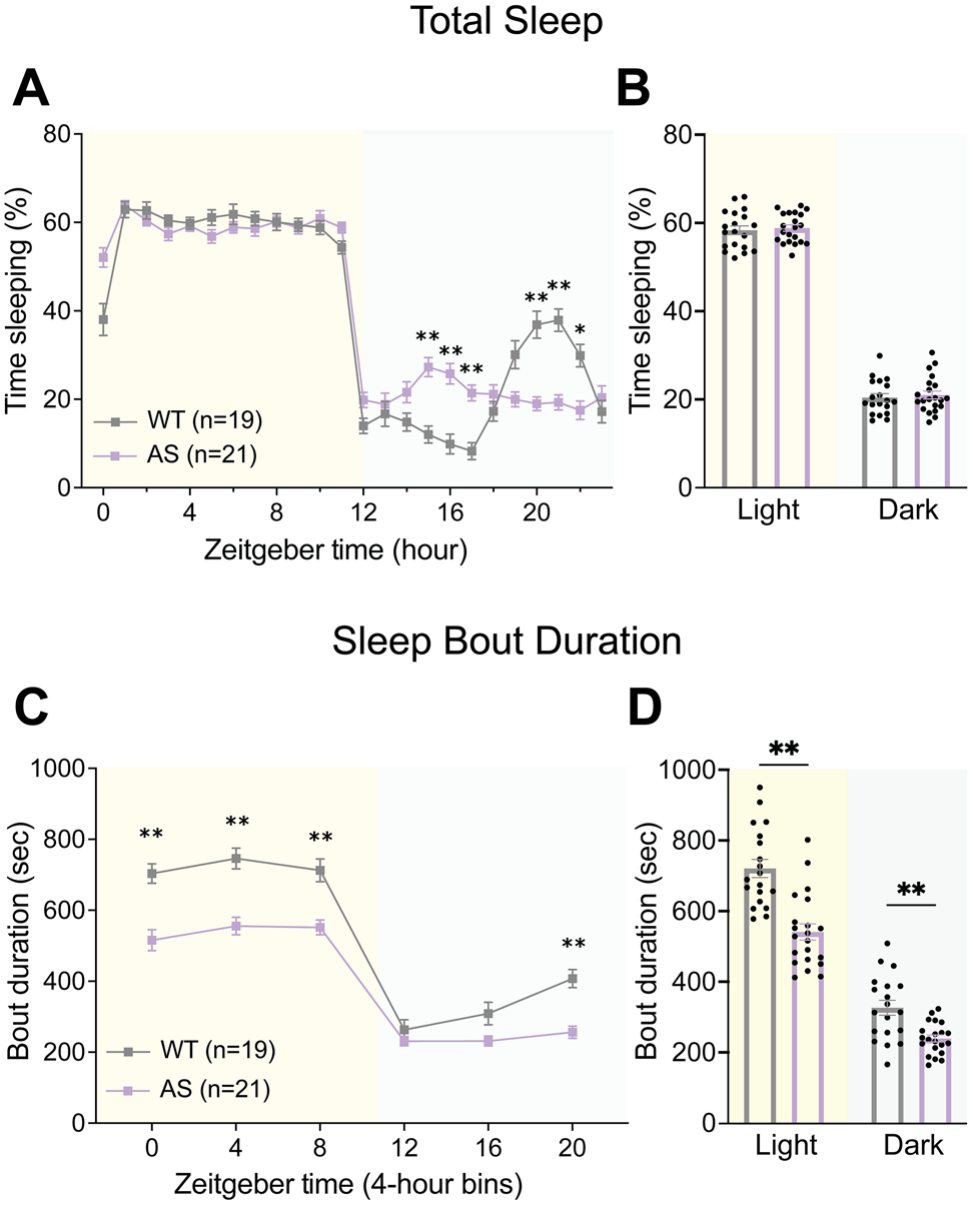
AS mice exhibit altered sleep patterns. ***A***, Piezoelectric quantification of hourly percent sleep in AS and WT mice. ***B***, Average percent sleep across light and dark cycles. ***C***, Mean sleep bout durations across 4 h time bins. ***D***, Mean sleep bout duration during light and dark cycle, averaged from 4 h bins. All panels analyzed using two-way RM ANOVA with Šídák's post hoc tests. Data presented as mean ± SEM. *p < 0.05, **p < 0.01. Individual data points labeled by genotype and sex are reported in Extended Data Figure 4-1.

**Figure 5.**
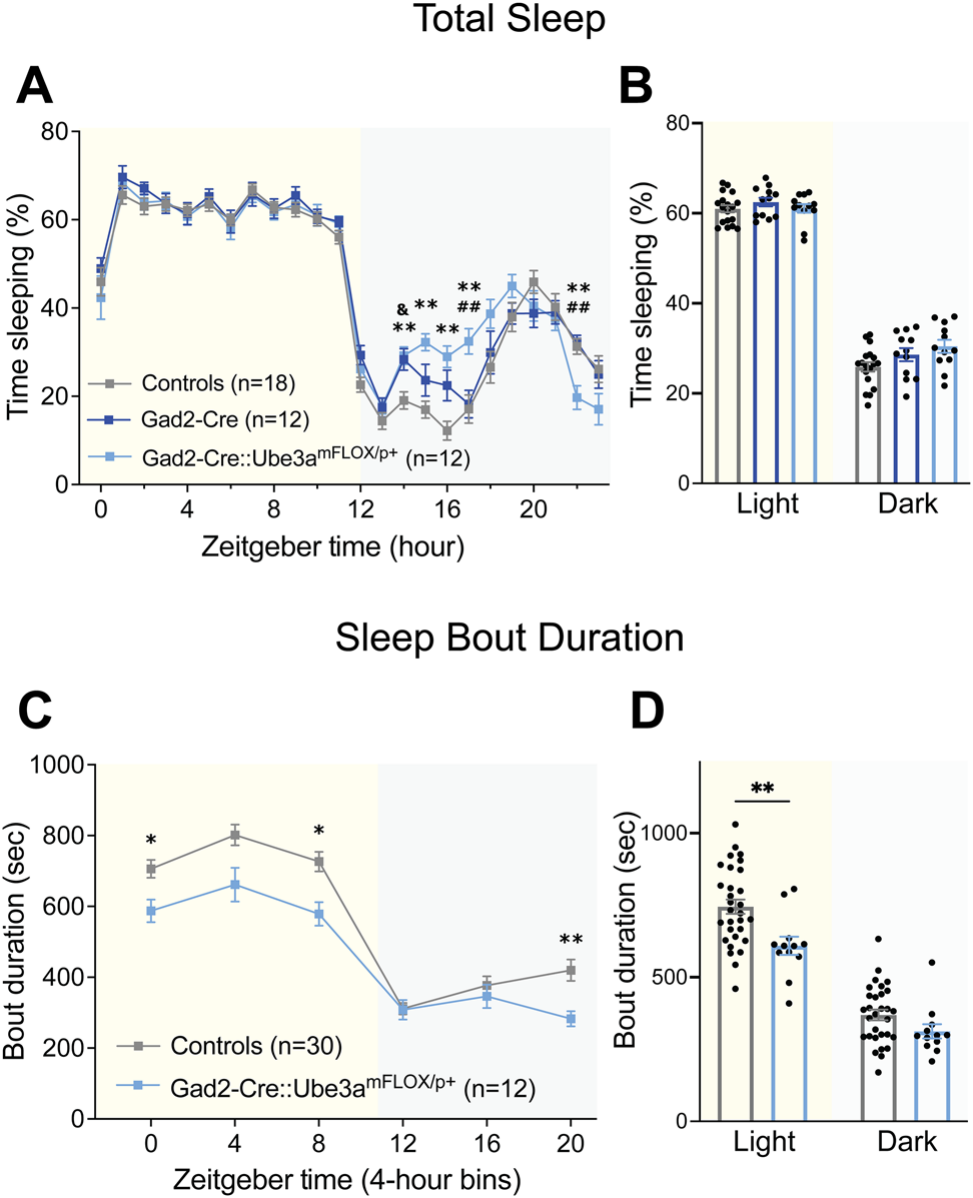
Gad2-Cre::*Ube3a^mFLOX/p+^* mice exhibit fragmented sleep. ***A***, Piezoelectric quantification of hourly percent sleep in Cre-negative controls, Gad2-Cre controls, and Gad2-Cre::*Ube3a^mFLOX/p+^* mice. ***B***, Average percent sleep across light and dark cycles. ***C***, Mean sleep bout durations across 4-hour time bins in Controls and Gad2-Cre::*Ube3a^mFLOX/p+^* mice. ***D***, Mean sleep bout duration during light and dark cycle, averaged from 4 h bins. All panels analyzed using two-way RM ANOVA with Šídák's post hoc tests. Data presented as mean ± SEM. *Cre-negative controls versus Gad2-Cre::*Ube3a^mFLOX/p+^*, ^#^Gad2-Cre controls versus Gad2-Cre::*Ube3a^mFLOX/p+^*, ^&^Cre-negative controls versus Gad2-Cre controls. *p < 0.05, **p < 0.01. Individual data points labeled by genotype and sex are reported in Extended Data Figure 5-1.

**Figure 6.**
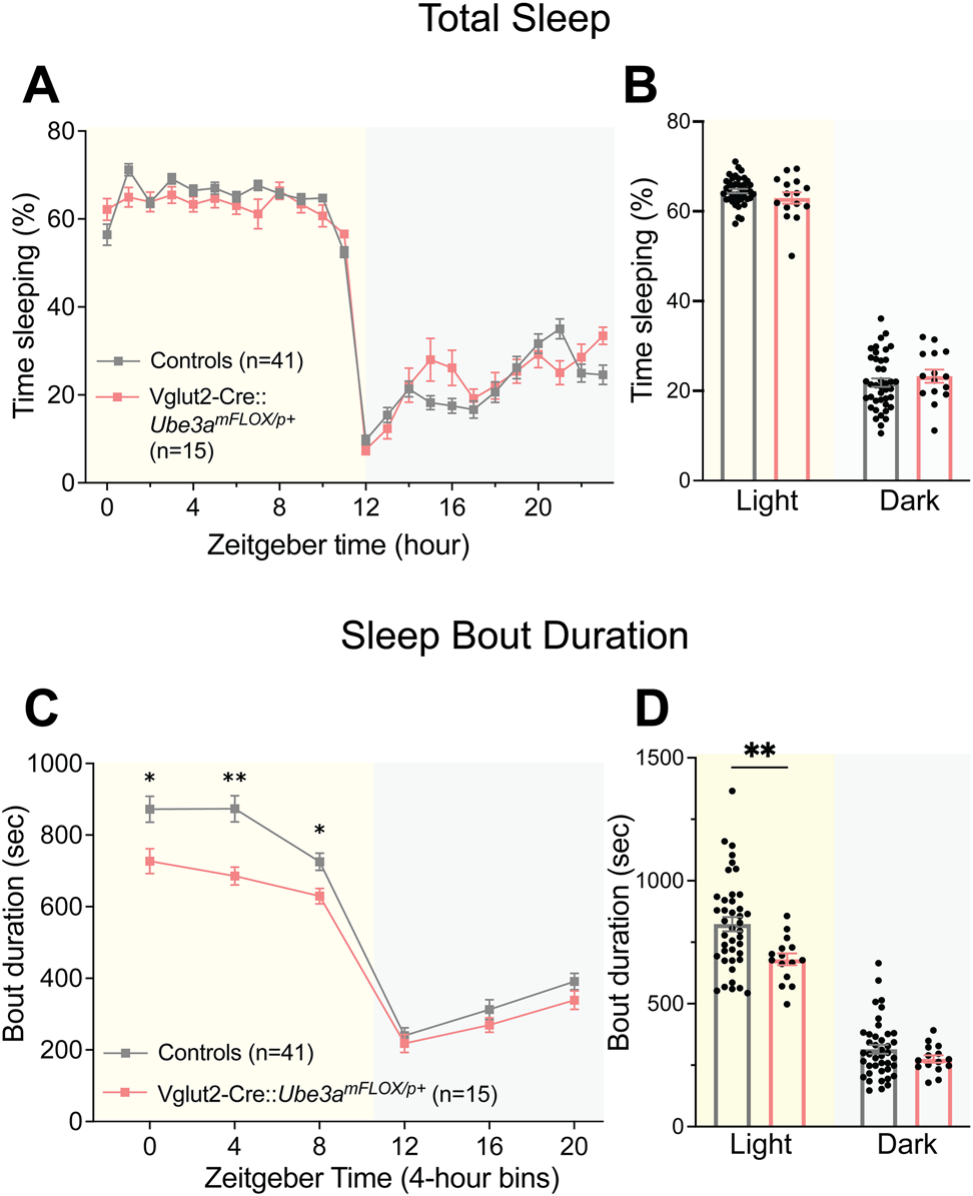
Vglut2-Cre::*Ube3a^mFLOX/p+^* mice exhibit altered sleep behavior. ***A***, Piezoelectric quantification of hourly percent sleep in Vglut2-Cre::*Ube3a^mFLOX/p+^* mice and controls. ***B***, Average percent sleep across light and dark cycles. ***C***, Mean sleep bout durations across 4 h time bins. ***D***, Mean sleep bout duration during light and dark cycle, averaged from 4 h bins. All panels analyzed using two-way RM ANOVA with Šídák's post hoc tests. Data presented as mean ± SEM. *p < 0.05, **p < 0.01. Individual data points labeled by genotype and sex are reported in Extended Data Figure 6-1.

**Figure 7.**
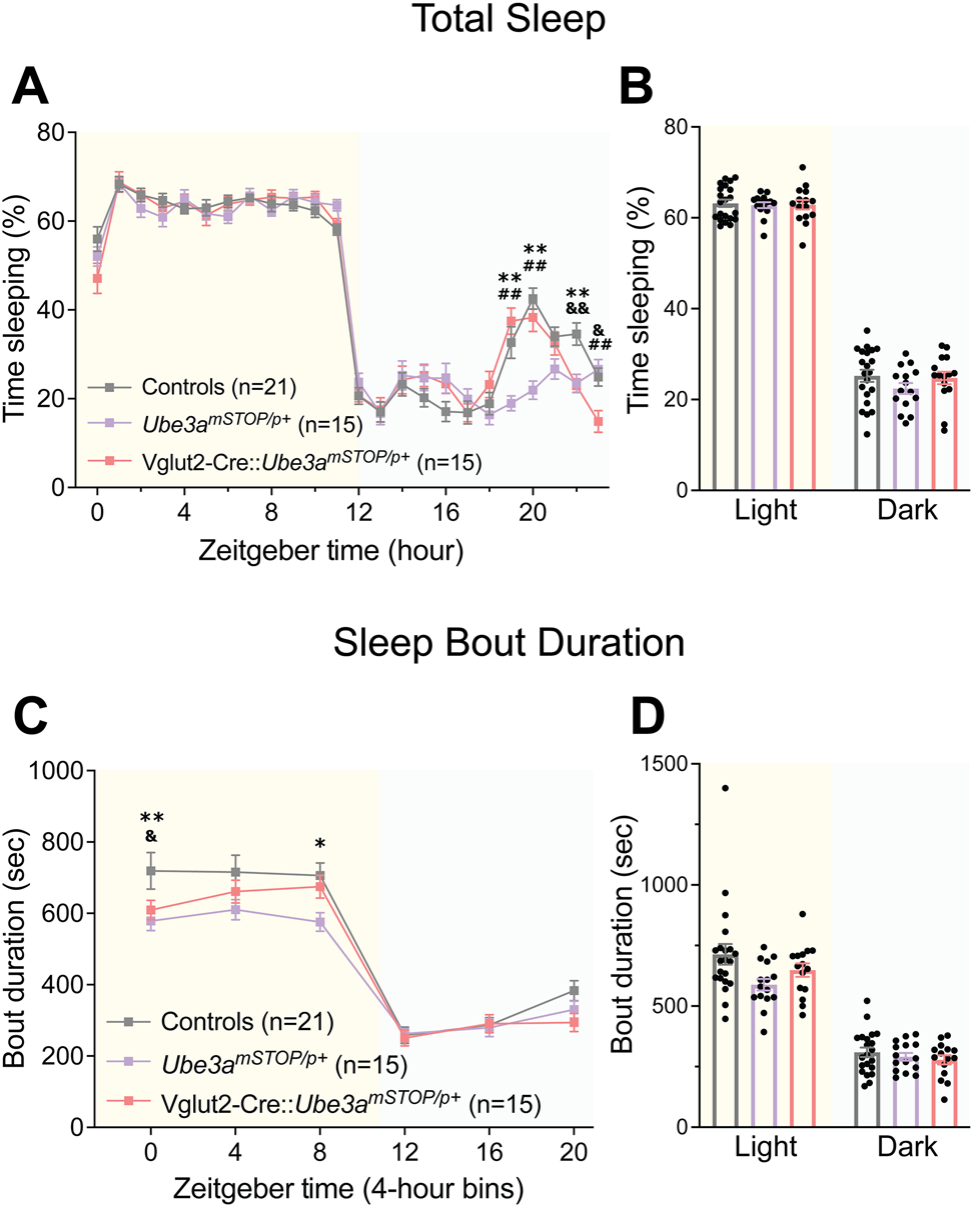
Glutamatergic neuron-selective reinstatement of *Ube3a* rescues sleep patterns in AS model mice. ***A***, Piezoelectric quantification of hourly percent sleep in Vglut2-Cre::*Ube3a^mSTOP/p+^*, *Ube3a^mSTOP/p+^*, and control mice. ***B***, Average percent sleep across light and dark cycles. ***C***, Mean sleep bout durations across 4 h time bins. ***D***, Mean sleep bout duration during light and dark cycle, averaged from 4 h bins. All panels analyzed using two-way RM ANOVA with Šídák's post hoc tests. Data presented as mean ± SEM. *Controls versus *Ube3a^mSTOP/p+^*, ^#^*Ube3a^mSTOP/p+^* versus Vglut2-Cre::*Ube3a^mSTOP/p+^*, ^&^Controls versus Vglut2-Cre::*Ube3a^mSTOP/p+^*. *p < 0.05, **p < 0.01. Individual data points labeled by genotype and sex are reported in Extended Data Figure 7-1.

**Figure 8.**
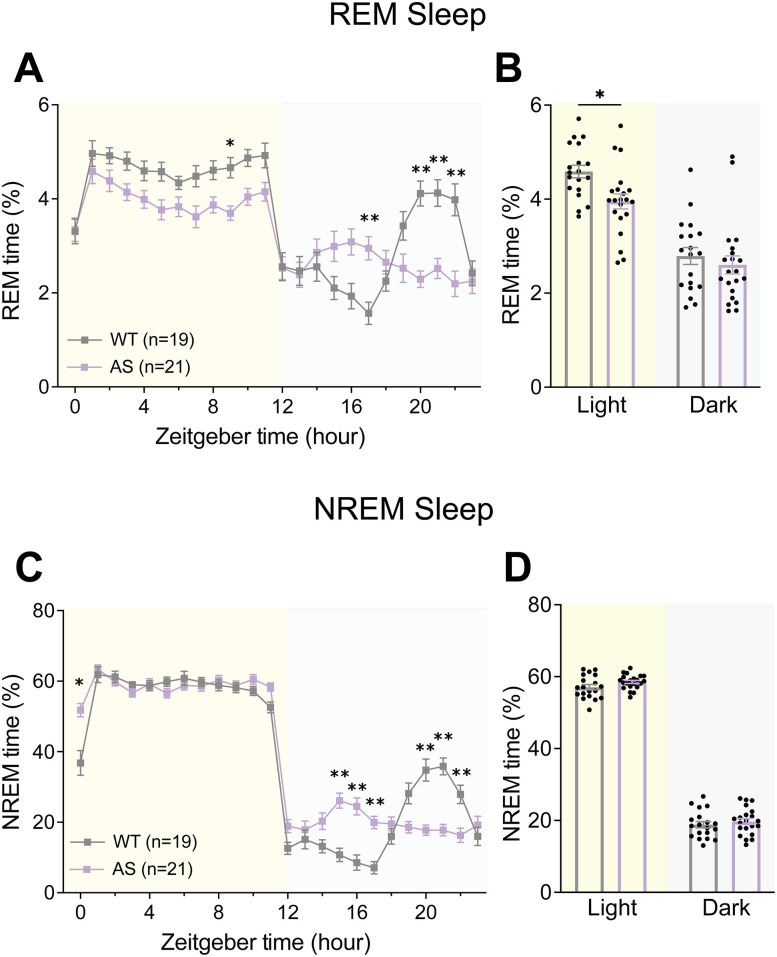
AS model mice exhibit decreased REM sleep. ***A***, Piezoelectric estimation of hourly percent REM sleep in AS and WT mice. ***B***, Average percent REM across light and dark cycles. ***C***, Estimated hourly percent NREM sleep in AS and WT mice. ***D***, Average percent NREM across light and dark cycles. All panels analyzed using two-way RM ANOVA with Šídák's post hoc tests. Data presented as mean ± SEM. *p < 0.05, **p < 0.01. Individual data points labeled by genotype and sex are reported in Extended Data Figure 8-1.

**Figure 9.**
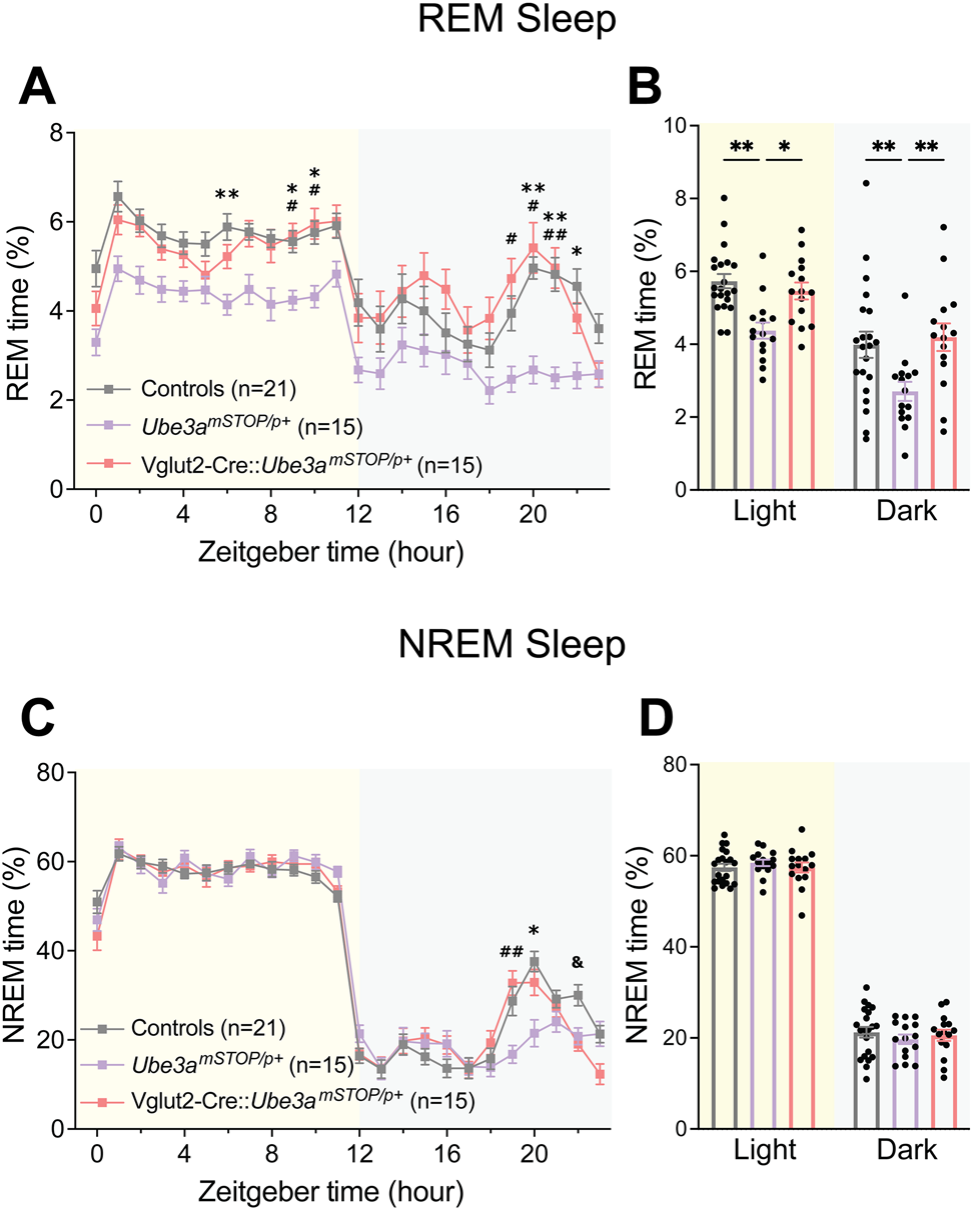
Glutamatergic neuron-selective reinstatement of *Ube3a* normalizes sleep composition. ***A***, Piezoelectric estimation of hourly percent REM sleep in Vglut2-Cre::*Ube3a^mSTOP/p+^*, *Ube3a^mSTOP/p+^*, and control mice. ***B***, Average percent REM across light and dark cycles. ***C***, Estimated hourly percent NREM sleep in AS and WT mice. ***D***, Average percent NREM across light and dark cycles. All panels analyzed using two-way RM ANOVA with Šídák's post hoc tests. Data presented as mean ± SEM. *Controls versus *Ube3a^mSTOP/p+^*, ^#^*Ube3a^mSTOP/p+^* versus Vglut2-Cre::*Ube3a^mSTOP/p+^*, ^&^Controls versus Vglut2-Cre::*Ube3a^mSTOP/p+^*. *p < 0.05, **p < 0.01. Individual data points labeled by genotype and sex are reported in Extended Data Figure 9-1 (REM sleep estimates) and Extended Data Figure 9-2 (NREM sleep estimates).

1. Two-group open field distance traveled, rotarod, nest building (Figs. 1, 2): Two-way repeated-measures ANOVA
2. Three-group open field distance traveled, rotarod, nest building (Fig. 3): Two-way repeated-measures ANOVA with Tukey's post hoc multiple comparisons for main effects of genotype
3. Two-group marble burying and open field center time (Fig. 1*E*): Unpaired, two-tailed *t* test
4. Three-group marble burying (Figs. 2*D*, 3*B*): Brown–Forsythe ANOVA with Dunnett's T3 multiple comparisons
5. Three-group open field center time (Extended Data Fig. 3-2*B*): One-way ANOVA
6. Sleep studies (Figs. 4–9): Two-way repeated-measures ANOVA with Šidák’s multiple-comparisons tests

Data are presented as mean ± SEM unless otherwise noted. A *p*-value <0.05 was considered statistically significant. GraphPad Prism 10.3.1 software was used for all statistical analyses. Tukey post hoc tests were used when all comparisons were evaluated (overall comparisons between each genotype). Šidák’s post hoc tests were used for sleep analyses in which post hoc comparisons were only used to assess for differences between groups within the same time point (no comparisons were being made across different time points). Brown–Forsythe ANOVA and Dunnett's T3 multiple-comparisons tests were used when genotypic groups displayed unequal variances as measured by the Brown–Forsythe test. The statistical tests used for each figure and the statistical outputs are reported in Extended Data Figure 1-2.

Sample sizes required for appropriate statistical power were based on a meta-analysis of AS mouse model behavioral tasks (Sonzogni et al., 2018) and previous behavioral studies of AS mice (Sonzogni et al., 2019; Judson et al., 2021).

## Results

### Deletion of *Ube3a* in GABAergic neurons yields few deficits in a battery of AS-phenotypic behaviors

To assess the role of GABAergic neurons in AS-related behaviors, we generated mice with maternal inheritance of a floxed *Ube3a* allele and paternal inheritance of Gad2-Cre, thereby deleting *Ube3a* from the majority of GABAergic neurons (Taniguchi et al., 2011; Judson et al., 2016; Fig. 1*A*). This genetic cross efficiently lowered UBE3A expression in inhibitory neurons, while leaving excitatory neuron UBE3A expression intact (Fig. 1*B*). We then tested these Gad2-Cre::*Ube3a^mFLOX/p+^* mice, alongside littermate controls, in a well-characterized behavioral battery in which AS mice exhibit reliable, robust deficits (Fig. 1*C*; Born et al., 2017; Sonzogni et al., 2018; Judson et al., 2021). For behavioral tests, we made the a priori decision to combine all control groups for statistical analysis. However, we unexpectedly detected a significant effect of Cre expression in some instances and thus reported these groups separately in those cases.

10.1523/ENEURO.0453-24.2025.f1-1 Figure 1-1 Gad2-Cre::*Ube3a^mFLOX/p^* *^+^* *­* behavioral battery labeled by genotype and sex. Open circles = males, closed circles = females. Dark gray = WT, light gray = *Ube3a^mFLOX/p+^*, dark blue = Gad2-Cre, light blue = Gad2-Cre::*Ube3a^mFLOX/p^* *^+^* . (**A**) Distance traveled in the open field across 5-minute bins. (**B**) Total time in the center of the open field. (**C**) Quantification of marble burying behavior by manual count of buried marbles (left panel) and threshold-based analysis (right panel). (**D**) Latency to fall or first passive rotation on the rotarod across each acquisition (day 1) and retest (day 2) trial. (**E**) Quantification of percent nesting material used across 5-day test. (**F**) Nest building behavior from only maternal heterozygous *Ube3a^m+/pFLOX^* litters with additional cohort added. WT and *Ube3a^mFLOX/p+^* controls (n = 7), Gad2-Cre (n = 8), Gad2-Cre::*Ube3a^mFLOX/p+^* (n = 10). Two-way RM ANOVA with Tukey’s post hoc comparisons for effect of genotype. Data presented as means ± SEM. *P < 0.05, **P < 0.01. Download Figure 1-1, TIF file.

10.1523/ENEURO.0453-24.2025.f1-2 Figure 1-2 **Statistical table.** Summary table of statistical tests and outputs by figure. Download Figure 1-2, XLS file.

Mice were first tested in the open field test, a measure of locomotor ability and exploration behavior in which AS model mice are hypoactive. Gad2-Cre::*Ube3a^mFLOX/p+^* mice displayed no significant deficits in this behavioral test, suggesting no overt motor impairment (Fig. 1*D*; main effect of genotype: *F*_(1,41)_ = 0.7491, *p* = 0.3918). These mice also showed no difference in time spent in the center of the arena, suggesting no difference in anxiety-like behavior (Extended Data Fig. 1-1*B*; *t*_(41)_ = 1.402, *p* = 0.1684). Mice were next tested in the marble burying assay, a test evaluating motor coordination and the innate digging behavior of mice (Thomas et al., 2009). In this behavior, Gad2-Cre::*Ube3a^mFLOX/p+^* mice again performed similarly to controls, using an unbiased thresholding analysis to quantify the marble area unobscured by bedding (Fig. 1*E*; *t*_(40)_ = 0.7272, *p* = 0.4713; Mossner et al., 2020; Judson et al., 2021).

Mice were next tested in a 2 d variation of the rotarod test assessing motor coordination and learning. In accordance with their having typical open field and marble burying performance, Gad2-Cre::*Ube3a^mFLOX/p+^* mice performed similarly to controls in the rotarod task (Fig. 1*F*; main effect of genotype: *F*_(1,39)_ = 2.569, *p* = 0.1171), although they showed a tendency toward decreased latency to fall or passively rotate on the retest day.

After displaying only minor deficits in three largely motor tasks, mice were single-housed and assessed for nest building behavior, an innate, presleep behavioral program performed by male and female mice for protection, thermoregulation, and parenting (Sotelo et al., 2022; Tagawa et al., 2024). In contrast to the previous behavioral tests, Gad2-Cre::*Ube3a^mFLOX/p+^* mice exhibited a significant decrease in nest material used, indicating a behavioral deficit similar to AS model mice (Fig. 1*G*; main effect of genotype: *F*_(1,37)_ = 20.28, *p* < 0.0001). Since the majority of control mice used for this experiment were *Ube3a^mFLOX/p+^* mice, due to breeding with *Ube3a^mFLOX/pFLOX^* females, we repeated the nest building assay with an additional cohort of mice from *Ube3a^m+/pFLOX^* females to ensure this deficit was not due to the Gad2-Cre allele alone. When all litters from heterozygous crosses were combined, Gad2-Cre mice performed similarly to Cre-negative controls, and Gad2-Cre::*Ube3a^mFLOX/p+^* mice used significantly less nest material than Gad2-Cre controls (Extended Data Fig. 1-1*F*; main effect of genotype: *F*_(2,22)_ = 8.711, *p* = 0.0016, post hoc tests: Gad2-Cre vs Gad2-Cre::*Ube3a^mFLOX/p+^*: *q*_(22)_ = 4.889, *p* = 0.0061). Taken together, these data suggest that, unlike its documented role in EEG activity and seizure susceptibility (Judson et al., 2016; Gu et al., 2019), *Ube3a* loss from GABAergic neurons is not the driver of many other known AS mouse behavioral phenotypes, excepting nest building behavior.

### Glutamatergic deletion of *Ube3a* produces multiple behavioral deficits

Given that *Ube3a* loss in GABAergic neurons yielded few behavioral deficits, we reasoned that *Ube3a* deletion from glutamatergic neurons might instead drive many AS behavioral phenotypes. Accordingly, we generated mice with maternal inheritance of the floxed *Ube3a* construct and paternal inheritance of Vglut2-Cre (*Slc17a6*; Vong et al., 2011). While VGLUT2 is primarily expressed subcortically in adulthood, it is expressed broadly in the cortex and hippocampus during development, allowing us, among other groups, to use this line to broadly target glutamatergic neurons (Meng et al., 2016; Gonzalez et al., 2019; Kim et al., 2024). Indeed, Vglut2-Cre-mediated deletion of *Ube3a* efficiently lowered UBE3A expression in most glutamatergic neurons, while sparing inhibitory neuron UBE3A expression (Fig. 2*A*,*B*).

10.1523/ENEURO.0453-24.2025.f2-1 Figure 2-1 **Vglut2-Cre::*Ube3a^mFLOX/p^*** ***^+^*** ***­* behavioral battery labeled by genotype and sex.** Open circles = males, closed circles = females. Dark gray = WT, light gray = *Ube3a^mFLOX/p+^*, dark red = Vglut2-Cre, light red = Vglut2-Cre::*Ube3a^mFLOX/p^* *^+^* . (**A**) Distance traveled in the open field across 5-minute bins. (**B**) Total time in the center of the open field. (**C**) Quantification of marble burying behavior by manual count of buried marbles (left panel) and threshold-based analysis (right panel). (**D**) Latency to fall or first passive rotation on the rotarod across each acquisition (day 1) and retest (day 2) trial. (**E**) Quantification of percent nesting material used across 5-day test. Download Figure 2-1, TIF file.

In contrast to mice with GABAergic *Ube3a* deletion, Vglut2-Cre::*Ube3a^mFLOX/p+^* mice showed significantly decreased distance traveled in the open field test (Fig. 2*C*; main effect of genotype: *F*_(1,45)_ = 6.053, *p* = 0.0178). Despite their decreased locomotor performance, these mice showed no difference in time spent in the center of the arena (Extended Data Fig. 2-1*B*; *t*_(45)_ = 0.2252, *p* = 0.8228). When tested in the marble burying task, Vglut2-Cre::*Ube3a^mFLOX/p+^* mice also showed a striking decrease in burying behavior (Fig. 2*D*; main effect of genotype: *F*_(2,33.49)_ = 28.05, *p* < 0.0001). Notably, Vglut2-Cre mice showed an intermediate marble burying deficit when compared with Cre-negative littermates (Fig. 2*D*; *t*_(30.99)_ = 3.335, *p* = 0.0066). Nonetheless, Vglut2-Cre::*Ube3a^mFLOX/p+^* mice displayed significantly impaired burying when compared with Vglut2-Cre controls (Fig. 2*D*; *t*_(13.28)_ = 4.219, *p* = 0.0029), suggesting a key role for *Ube3a* loss in glutamatergic neurons for this phenotype.

Similarly to the first two behaviors tested, Vglut2-Cre::*Ube3a^mFLOX/p+^* mice showed significantly impaired performance on the rotarod task (Fig. 2*E*; main effect of genotype: *F*_(1,45)_ = 6.897, *p* = 0.0118). Lastly, Vglut2-Cre::*Ube3a^mFLOX/p+^* mice were tested in the nest building assay, in which they showed comparable performance to littermate controls (Fig. 2*F*; main effect of genotype: *F*_(1,44)_ = 1.397, *p* = 0.2436). Together, these data suggest glutamatergic neuron loss of UBE3A drives several behaviors, particularly motor behaviors in AS model mice, while loss of UBE3A from GABAergic neurons plays a larger role in regulating nest building behavior.

### Reinstatement of *Ube3a* in glutamatergic neurons rescues multiple AS behavioral phenotypes

To further probe and validate the role of glutamatergic deletion of *Ube3a* in AS-associated behaviors, we utilized the *Ube3a^mSTOP/p+^* model of *Ube3a* reinstatement (Silva-Santos et al., 2015), crossing female *Ube3a^m+/pSTOP^* mice with male Vglut2-Cre mice. This cross generated (1) mice with pan-neuronal deletion of *Ube3a* (*Ube3a^mSTOP/p+^*, equivalent to AS model mice), (2) mice with *Ube3a* reinstatement in glutamatergic neurons (Vglut2-Cre::*Ube3a^mSTOP/p+^*), and (3) littermate controls, all of which were tested in the same battery of behavioral tests. Vglut2-Cre-mediated *Ube3a* reinstatement substantially restored UBE3A expression in glutamatergic neurons, while leaving GABAergic neurons devoid of UBE3A (Extended Data Fig. 3-1*A,B*).

When assessed in the open field, Vglut2-Cre::*Ube3a^mSTOP/p+^* mice unexpectedly showed comparable distance traveled to *Ube3a^mSTOP/p+^* mice, indicating no rescue in behavioral performance (Fig. 3*A*; main effect of genotype: *F*_(2,59)_ = 15.53, *p* < 0.0001, post hoc tests: Controls vs *Ube3a^mSTOP/p+^*: *q*_(59)_ = 6.335, *p* = 0.0001, Controls vs Vglut2-Cre::*Ube3a^mSTOP/p+^*: *q*_(59)_ = 6.661, *p* < 0.0001, *Ube3a^mSTOP/p+^* vs Vglut2-Cre::*Ube3a^mSTOP/p+^*: *q*_(59)_ = 6.661, *p* = 0.8988). This suggests that while glutamatergic deletion of *Ube3a* is sufficient to drive a behavioral deficit in this test, this neuronal population likely does not drive this behavior alone. Interestingly, neither *Ube3a^mSTOP/p+^* nor Vglut2-Cre::*Ube3a^mSTOP/p+^* mice showed a robust deficit in time spent in the center of the area, consistent with other studies demonstrating absent or weak anxiety-like phenotypes of AS model mice (Extended Data Fig. 3-2*B*; *F*_(2,59)_ = 1.347, *p* = 0.2680; Born et al., 2017; Syding et al., 2022; Tanas et al., 2022). In contrast, when tested in the marble burying test, Vglut2-Cre::*Ube3a^mSTOP/p+^* mice showed significantly improved performance compared with *Ube3a^mSTOP/p+^* mice, and similar performance to that of littermate controls (Fig. 3*B*; *F*_(2,42.73)_ = 13.7, *p* < 0.0001, post hoc tests: *Ube3a^mSTOP/p+^* vs Vglut2-Cre::*Ube3a^mSTOP/p+^*: *t*_(16.55)_ = 3.630, *p* = 0.0061, Controls vs Vglut2-Cre::*Ube3a^mSTOP/p+^*: *t*_(35.44)_ = 1.355, *p* = 0.4500). This experiment was not sufficiently powered to detect the previously observed difference between Vglut2-Cre controls and WT mice; however, Cre*^+^* mice tended to bury fewer marbles on average in this experiment (Extended Data Fig. 3-2*C*).

10.1523/ENEURO.0453-24.2025.f3-1 Figure 3-1 **Glutamatergic neuron *Ube3a* reinstatement in Vglut2-Cre::*Ube3a^mSTOP/p+^* mice.** (**A**) UBE3A immunostaining in Control, *Ube3a^mSTOP/p+^* and Vglut2-Cre::*Ube3a^mSTOP/p+^* mice. Asterisks indicate excitatory (NeuN+, GAD-) cells. Zoom images demonstrate cell type-specific UBE3A expression in the somatosensory cortex. Arrows indicate inhibitory (NeuN+, GAD+) neurons. Arrowheads indicate non-neuronal (NeuN-) cells with persistent, weak UBE3A expression in *Ube3a^mSTOP/p+^* mice. Hemi-section scale bar = 1 mm. Zoom image scale bar = 10 µm. (**B**) Nuclear UBE3A intensity of individual excitatory (NeuN+, GAD-) and inhibitory (NeuN+, GAD+) cells in the somatosensory cortex displayed as violin plots with means ± SD. Download Figure 3-1, TIF file.

10.1523/ENEURO.0453-24.2025.f3-2 Figure 3-2 **Vglut2-Cre::*Ube3a^mSTOP/p+^* behavioral battery labeled by genotype and sex.** Open circles = males, closed circles = females. Gray = WT, dark red = Vglut2-Cre, purple = *Ube3a^mSTOP/p+^*, light red = Vglut2-Cre::*Ube3a^mSTOP/p^* *^+^* . (**A**) Distance traveled in the open field across 5-minute bins. (**B**) Total time in the center of the open field. (**C**) Quantification of marble burying behavior by manual count of buried marbles (left panel) and threshold-based analysis (right panel). (**D**) Latency to fall or first passive rotation on the rotarod across each acquisition (day 1) and retest (day 2) trial. (**E**) Quantification of percent nesting material used across 5-day test. Download Figure 3-2, TIF file.

In the rotarod task, Vglut2-Cre::*Ube3a^mSTOP/p+^* mice showed comparable motor performance to controls, representing a full phenotypic rescue (Fig. 3*C*; main effect of genotype: *F*_(2,59)_ = 17.84, *p* < 0.0001, post hoc: *Ube3a^mSTOP/p+^* vs Vglut2-Cre::*Ube3a^mSTOP/p+^*: *q*_(59)_ = 5.730, *p* = 0.0004). Finally, when evaluated in the nest building task, Vglut2-Cre::*Ube3a^mSTOP/p+^* mice demonstrated a partial phenotypic rescue (Fig. 3*D*; main effect of genotype: *F*_(2,57)_ = 34.46, *p* < 0.0001, post hoc: *Ube3a^mSTOP/p+^* vs Vglut2-Cre::*Ube3a^mSTOP/p+^*: *q*_(57)_ = 4.962, *p* = 0.0025, Controls vs Vglut2-Cre::Ube3amSTOP/p+ mice: q(57) = 5.430, p = 0.0009), suggesting a partial role for glutamatergic neuron loss of UBE3A in this behavior. Overall, these data suggest that UBE3A loss in glutamatergic neurons plays a major role in the AS-related motor and innate behaviors studied, while fewer behaviors are impacted by UBE3A loss from GABAergic neurons.

### AS mice exhibit altered sleep–wake behavior

Sleep disturbances, such as frequent nighttime awakenings and difficulty falling asleep, are common in individuals with AS (Goldman et al., 2012; Pereira et al., 2020; Qu et al., 2024); however, the mechanisms underlying sleep deficits in AS are poorly understood. As GABAergic circuitry is critical for the onset and maintenance of sleep, likely through the inhibition of wake-promoting centers in the brainstem and hypothalamus (Anaclet et al., 2014; Scammell et al., 2017; Takata et al., 2018; Sulaman et al., 2023), we speculated that the loss of UBE3A from GABAergic neurons might be particularly impactful for AS sleep phenotypes. Furthermore, AS individuals and model mice demonstrate increased delta EEG power (Sidorov et al., 2017; den Bakker et al., 2018; Copping and Silverman, 2021), which has been linked to altered sleep (Huber et al., 2000; Tononi and Cirelli, 2006; Hubbard et al., 2020), and this delta phenotype is largely mediated by GABAergic neuron loss of *Ube3a* (Judson et al., 2016).

Alterations in sleep patterns and composition have been well-studied in the *Ube3a^m−/p+^* mouse model of AS using EEG/EMG recordings (Ehlen et al., 2015; Shi et al., 2015; Lee et al., 2023). These studies, however, are exceptionally labor-intensive and therefore not ideally suited for screening sleep behavior in numerous genetic crosses. Thus, we sought to determine whether sleep phenotypes of AS model mice could be replicated using the PiezoSleep monitoring system, a noninvasive piezoelectric system that uses breathing patterns to monitor sleep in single-housed mice. This technology has been validated with simultaneous EEG/EMG measurements (Mang et al., 2014) and has reproduced sleep phenotypes found using EEG/EMG measurements in a mouse model of ASD (Lord et al., 2022). To evaluate the utility of this technology for our study, AS mice and littermate controls were single-housed in piezoelectric chambers and allowed to acclimate for two dark cycles followed by 7–9 d of data collection. Sleep behavior was quantified by averaging data across days for each animal, providing a robust measurement of daily sleep patterns.

Consistent with previous reports of C57BL/6J mouse behavior, wild-type mice demonstrated a robust sleep period in the latter half of the dark cycle, termed the “siesta” (Wisor et al., 2008; Collins et al., 2020; Fig. 4*A*, Extended Data Fig. 4-1*A,E*). AS mice, on the other hand, virtually lacked this behavior, showing relatively stable percent sleep across the dark cycle, as previously described (Ehlen et al., 2015; Shi et al., 2022; Fig. 4*A*; genotype × time interaction: *F*_(23,874)_ = 11.67, *p* < 0.0001). Despite this difference in sleep patterning, AS mice demonstrated comparable total sleep in the light and dark phases (Fig. 4*B*; main effect of genotype: *F*_(1,38)_ = 0.2925, *p* = 0.5918).

10.1523/ENEURO.0453-24.2025.f4-1 Figure 4-1 **AS mouse sleep behavior separated by sex.** Open circles = males, closed circles = females. (**A**) Piezoelectric quantification of hourly percent sleep in male and female AS and WT mice. (**B**) Average percent sleep across light and dark cycles. (**C**) Mean sleep bout durations across 4-hour time bins. (**D**) Mean sleep bout duration during light and dark cycle, averaged from 4-hour bins. (**E**) Piezoelectric quantification of hourly percent sleep presented in minutes. (**F**) Average percent sleep across light and dark cycles presented in minutes. Data presented as means ± SEM. Download Figure 4-1, TIF file.

We then quantified mean sleep bout duration, used as a measure of sleep fragmentation. Interestingly, AS model mice showed a significant reduction in mean sleep bout duration across the light cycle and at the end of the dark cycle (Fig. 4*C*; main effect of genotype: *F*_(1,38)_ = 31.41, *p* < 0.0001), corresponding to overall decreased mean bout length in both light and dark phases (Fig. 4*D*; post hoc: light: *t*_(76)_ = 6.200, *p* < 0.0001, dark: *t*_(76)_ = 2.998, *p* = 0.0073). As AS mice show no notable difference in total sleep in the light or dark cycles, this decreased mean sleep bout length provides evidence for increased fragmentation of sleep, resembling the nighttime awakenings and decreased sleep efficiency seen in AS individuals (Trickett et al., 2019; O’Rourke et al., 2024).

### GABAergic deletion of *Ube3a* increases sleep fragmentation

We next assessed the sleep behavior of Gad2-Cre::*Ube3a^mFLOX/p+^* mice to determine whether the sleep phenotypes observed in AS mice are due to GABAergic neuron deletion of *Ube3a*. On analysis of hourly sleep patterns, Gad2-Cre::*Ube3a^mFLOX/p+^* mice, surprisingly, showed intact “siesta” behavior, although they exhibited a dark cycle sleeping period that was shifted slightly earlier than controls; this effect may be partially due to a slightly different sleep pattern of the Gad2-Cre control mice (Fig. 5*A*; Extended Data Fig. 5-1*A,C*; genotype × time interaction: *F*_(46,897)_ = 3.040, *p* < 0.0001). Corresponding with the slightly earlier and lengthened “siesta” period, Gad2-Cre::*Ube3a^mFLOX/p+^* mice showed a trend toward increased total sleep in the dark cycle (Fig. 5*B*; genotype × time interaction: *F*_(2,39)_ = 3.164, *p* = 0.0533). These results suggest that GABAergic neuron loss of *Ube3a* does not play an important role in the loss of “siesta” in AS mice.

Upon analysis of sleep bout duration, however, Gad2-Cre::*Ube3a^mFLOX/p+^* mice showed decreased mean bout durations across much of the light cycle and the end of the dark cycle (Fig. 5*C*; main effect of genotype: *F*_(1,40)_ = 8.654, *p* = 0.0054), corresponding to an overall decrease in bout duration in the light cycle (Fig. 5*D*; post hoc: light cycle: *t*_(80)_ = 3.459, *p* = 0.0017, dark cycle: *t*_(80)_ = 1.454, *p* = 0.2771). Therefore, while GABAergic *Ube3a* deletion did not drive AS-like dysfunction in sleep pattern, GABAergic neurons appear responsible for at least part of the sleep fragmentation phenotype.

10.1523/ENEURO.0453-24.2025.f5-1 Figure 5-1 Gad2-Cre::*Ube3a^mFLOX/p^* *^+^* *­* sleep behavior separated by genotype and sex. Open circles = males, closed circles = females. Dark gray = WT, light gray = *Ube3a^mFLOX/p+^*, dark blue = Gad2-Cre, light blue = Gad2-Cre::*Ube3a^mFLOX/p^* *^+^* . (**A**) Piezoelectric quantification of hourly percent sleep in males. (**B**) Average percent sleep across light and dark cycles in males. (**C**) Hourly percent sleep in females. (**D**) Average percent sleep across light and dark cycles in females. (**E**) Mean sleep bout durations across 4-hour time bins. (**F**) Mean sleep bout duration during light and dark cycle, averaged from 4-hour bins. Data presented as means ± SEM. Download Figure 5-1, TIF file.

### Glutamatergic deletion of *Ube3a* disrupts sleep–wake behavior

To assess the role of glutamatergic neurons in AS sleep behavior, we tested Vglut2-Cre::*Ube3a^mFLOX/p+^* mice in the PiezoSleep chambers. Deletion of *Ube3a* from Vglut2-expressing neurons caused a slightly dysregulated hourly sleep pattern (Fig. 6*A*; Extended Data Fig. 6-1*A, C*; genotype × time interaction: *F*_(9.871, 533.1)_ = 2.634, *p* = 0.0040), though this effect was not as dramatic as the lack of “siesta” seen in AS mice. Despite this altered sleep pattern, Vglut2-Cre::*Ube3a^mFLOX/p+^* mice showed normal amounts of total sleep (Fig. 6*B*; main effect of genotype: *F*_(1,54)_ = 0.0002, *p* = 0.9644). Like AS model mice, Vglut2-Cre::*Ube3a^mFLOX/p+^* mice also showed decreased mean sleep bout duration (Fig. 6*C*; main effect of genotype: *F*_(1,54)_ = 7.309, *p* = 0.0092), with the most evident difference during the light cycle (Fig. 6*D*; post hoc: light cycle: *t*_(108)_ = 3.372, *p* = 0.0021, dark cycle: *t*_(108)_ = 0.9239, *p* = 0.5874). Similarly to the behavioral battery results, these data implicate glutamatergic neuron loss of *Ube3a* as an important driver of sleep dysfunction in AS mice.

10.1523/ENEURO.0453-24.2025.f6-1 Figure 6-1 Vglut2-Cre::*Ube3a^mFLOX/p^* *^+^* *­* sleep behavior separated by genotype and sex. Open circles = males, closed circles = females. Dark gray = WT, light gray = *Ube3a^mFLOX/p+^*, dark red = Vglut2-Cre, light red = Vglut2-Cre::*Ube3a^mFLOX/p^* *^+^* . (**A**) Piezoelectric quantification of hourly percent sleep in males. (**B**) Average percent sleep across light and dark cycles in males. (**C**) Hourly percent sleep in females. (**D**) Average percent sleep across light and dark cycles in females. (**E**) Mean sleep bout durations across 4-hour time bins. (**F**) Mean sleep bout duration during light and dark cycle, averaged from 4-hour bins. Data presented as means ± SEM. Download Figure 6-1, TIF file.

### Glutamatergic reinstatement of *Ube3a* rescues AS sleep patterning

To further assess the role of glutamatergic loss of *Ube3a* in AS-related sleep phenotypes, we next studied the sleep of mice with Vglut2-Cre-mediated *Ube3a* reinstatement (Vglut2-Cre::*Ube3a^mSTOP/p+^* mice). First, we confirmed that the *Ube3a^mSTOP/p+^* model of *Ube3a* deletion showed the same characteristic lack of “siesta” observed in AS model mice (Fig. 7*A*, Extended Data Fig. 7-1*A, C*). Interestingly, *Ube3a* reinstatement in this neuronal population was sufficient to induce a robust “siesta” behavior similar to that of controls (Fig. 7*A*; genotype × time interaction: *F*_(20.01, 480.2)_ = 3.959, *p* < 0.0001, post hoc: Vglut2-Cre::*Ube3a^mSTOP/p+^* vs *Ube3a^mSTOP/p+^*: hour 19: *t*_(22.28)_ = 5.420, *p* < 0.0001, hour 20: *t*_(24)_ = 4.344, *p* = 0.0007). Notably, this “siesta” in Vglut2-Cre::*Ube3a^mSTOP/p+^* mice terminated slightly earlier than controls, reminiscent of the sleep pattern of mice with GABAergic *Ube3a* deletion (post hoc: Controls vs Vglut2-Cre::*Ube3a^mSTOP/p+^*: hour 22: *t*_(31.69)_ = 3.968, *p* = 0.0012). This indicates that glutamatergic *Ube3a* reinstatement was sufficient to partially recover “siesta” behavior. As expected, Vglut2-Cre::*Ube3a^mSTOP/p+^* mice showed similar total sleep to controls and *Ube3a^mSTOP/p+^* mice (Fig. 7*B*; main effect of genotype: *F*_(2,48)_ = 0.9211, *p* = 0.4050).

10.1523/ENEURO.0453-24.2025.f7-1 Figure 7-1 Vglut2-Cre::*Ube3a^mSTOP/p^* *^+^* *­* sleep behavior separated by genotype and sex. Open circles = males, closed circles = females. Gray = WT, dark red = Vglut2-Cre, purple = *Ube3a^mSTOP/p+^*, light red = Vglut2-Cre::*Ube3a^mSTOP/p^* *^+^* . (**A**) Piezoelectric quantification of hourly percent sleep in males. (**B**) Average percent sleep across light and dark cycles. (**C**) Hourly percent sleep in females. (**D**) Average percent sleep across light and dark cycles in females. (**E**) Mean sleep bout durations across 4-hour time bins. (**F**) Mean sleep bout duration during light and dark cycle, averaged from 4-hour bins. Data presented as means ± SEM. Download Figure 7-1, TIF file.

On analysis of mean sleep bout duration, *Ube3a* reinstatement tended to increase bout duration toward the end of the light cycle (Fig. 7*C*; genotype × time interaction: *F*_(10,240)_ = 2.202, *p* = 0.0184); however, overall bout durations across the light and dark cycles did not reach statistical significance (Fig. 7*D*; main effect of genotype: *F*_(2,48)_ = 2.852, *p* = 0.0675, genotype × time interaction: *F*_(2,48)_ = 2.343, *p* = 0.1069). Taken together, these data suggest glutamatergic neurons play a major role in the sleep pattern of AS model mice, but only a partial role in sleep fragmentation.

### AS mice show decreased estimated REM sleep

In addition to sleep disturbance, AS individuals also show differences in sleep composition, namely, decreased REM sleep (Miano et al., 2004; Levin et al., 2022); mouse models of AS have likewise previously demonstrated a corresponding decrease in REM sleep during the light cycle (Colas et al., 2005; Lee et al., 2023). To determine whether the PiezoSleep chambers could be used to study sleep composition, we leveraged recent advancements in the analysis of piezoelectric signals to extract estimates of REM and NREM sleep based on changes in breathing rates characteristic of sleep state transitions (Mang et al., 2014; Yaghouby et al., 2016; Vanneau et al., 2021; Martin et al., 2024). In agreement with previous EEG/EMG studies (Colas et al., 2005; Lee et al., 2023), piezoelectric estimations of REM sleep revealed decreased REM in AS mice across the light cycle and dark cycle patterns mimicking the “siesta” phenotype seen in total sleep (Fig. 8*A*,*B*; Extended Data Fig. 8-1*A,B*; two-way ANOVA: main effect of genotype: *F*_(1,38)_ = 4.889, *p* = 0.0331, post hoc: light cycle: *t*_(76)_ = 2.675, *p* = 0.0182). Estimates of NREM sleep showed a similar lack of siesta in AS mice (Fig. 8*C*, Extended Data Fig. 8-1*C*; genotype × time interaction: *F*_(23,874)_ = 11.84, *p* < 0.0001) and a subtle, statistically nonsignificant increase in NREM sleep (Fig. 8*D*; main effect of genotype: *F*_(1,38)_ = 1.960, *p* = 0.1696).

10.1523/ENEURO.0453-24.2025.f8-1 Figure 8-1 **AS mouse estimated REM and NREM sleep separated by sex.** Open circles = males, closed circles = females. (**A**) Piezoelectric estimation of hourly percent REM sleep in male and female AS and WT mice. (**B**) Average percent REM across light and dark cycles. (**C**) Estimated hourly percent NREM sleep in AS and WT mice. (**D**) Average percent NREM across light and dark cycles. Data presented as means ± SEM. Download Figure 8-1, TIF file.

Given the rescue of sleep patterns by glutamatergic reinstatement of *Ube3a*, we predicted this would also rescue deficits of sleep composition. Indeed, Vglut2-Cre::*Ube3a^mSTOP/p+^* mice showed comparable REM sleep to controls across the light cycle (Fig. 9*A*, Extended Data Fig. 9-1*A*), demonstrating significantly increased light cycle REM time compared with *Ube3a^mSTOP/p+^* mice (Fig. 9*B*; main effect of genotype: *F*_(2,48)_ = 9.496, *p* = 0.0003, post hoc: *Ube3a^mSTOP/p+^* vs Vglut2-Cre::*Ube3a^mSTOP/p+^*: *t*_(96)_ = 2.499, *p* = 0.0418). Interestingly, we observed decreased REM in *Ube3a^mSTOP/p+^* mice during the dark cycle (post hoc: controls vs *Ube3a^mSTOP/p+^*: *t*_(96)_ = 3.180, *p* = 0.0059, *Ube3a^mSTOP/p+^* vs Vglut2-Cre::*Ube3a^mSTOP/p+^*: *t*_(96)_ = 3.421, *p* = 0.0028) that might be due to a trend toward increased REM sleep in the Vglut2-Cre controls (Extended Data Fig. 9-1*A–D*). Nonetheless, Vglut2-Cre::*Ube3a^mSTOP/p+^* mice were indistinguishable from Vglut2-Cre controls across the light cycle. Analysis of NREM estimates revealed a similar “siesta” phenotype seen in the total sleep trace that was largely rescued by glutamatergic reinstatement of *Ube3a* (Fig. 9*C*, Extended Data Fig. 9-2*A,C*), and total NREM sleep did not differ between groups (Fig. 9*D*; main effect of genotype: *F*_(2,48)_ = 0.05327, *p* = 0.9482). Overall, these findings support a critical role of glutamatergic neurons in the altered sleep composition seen in AS model mice.

10.1523/ENEURO.0453-24.2025.f9-1 Figure 9-1 Vglut2-Cre::*Ube3a^mSTOP/p^* *^+^* *­* REM sleep estimates separated by genotype and sex. (**A**) Piezoelectric estimation of hourly percent REM sleep in males. (**B**) Average percent REM across light and dark cycles in males. (**C**) Estimated hourly REM sleep in females. (**D**) Average percent REM across light and dark cycles in females. Data presented as means ± SEM. Download Figure 9-1, TIF file.

10.1523/ENEURO.0453-24.2025.f9-2 Figure 9-2 Vglut2-Cre::*Ube3a^mSTOP/p^* *^+^* *­* NREM sleep estimates separated by genotype and sex. (**A**) Piezoelectric estimation of hourly percent NREM sleep in males. (**B**) Average percent NREM across light and dark cycles in males. (**C**) Estimated hourly NREM sleep in females. (**D**) Average percent NREM across light and dark cycles in females. Data presented as means ± SEM. Download Figure 9-2, TIF file.

## Discussion

AS is a neurodevelopmental disorder with no disease-modifying treatment. However, clinical trials are currently underway using antisense oligonucleotides to unsilence the dormant paternal *UBE3A* allele, thereby normalizing UBE3A levels (Ionis: NCT05127226; Ultragenyx: NCT04259281). While this approach holds exciting promise and shows efficacy in mouse models (Meng et al., 2015; Milazzo et al., 2021), there is currently scant information regarding the key cell types or brain regions that require UBE3A reinstatement to mitigate core symptoms of AS. This holds particular importance, as effective biodistribution is a key concern in genetic therapies for CNS disorders (Roberts et al., 2020; Jafar-Nejad et al., 2021; Ling et al., 2023), and suboptimal targeting of necessary cell classes could hamper success. Moreover, mouse models of AS require early postnatal *Ube3a* reinstatement to achieve optimal phenotypic recovery (Silva-Santos et al., 2015; Sonzogni et al., 2020); early intervention could be difficult to achieve in the patient population without a corresponding early diagnosis, meaning many AS individuals are likely beyond the critical window to maximally benefit from *UBE3A* reinstatement-based therapies. Therefore, additional work is needed to better understand how loss of UBE3A leads to symptoms, as these insights will aid both in understanding the cell types that must be targeted for optimal genetic interventions and in developing alternative therapeutic options.

Our laboratory's previous work identified an outsized role of GABAergic loss of UBE3A in hyperexcitability phenotypes. GABAergic loss of UBE3A drives increased delta power on cortical EEG (Judson et al., 2016), a phenotype that correlates with the severity of a range of symptoms in AS individuals (Hipp et al., 2021; Ostrowski et al., 2021). Further, mice with *Ube3a* deleted from GABAergic neurons show decreased threshold to chemically and acoustically driven seizures, and they also exhibit spontaneous behavioral seizures, a phenotype not observed in AS model mice on a C57BL/6J background (Judson et al., 2016; Gu et al., 2019). These data forewarn that *UBE3A* reinstatement in a manner biased to glutamatergic neurons could potentially worsen epilepsy-related symptoms and highlight the importance of studying the neuronal populations regulating other behaviors.

Based on the exaggerated role of GABAergic neurons in AS seizure phenotypes, we predicted that GABAergic deletion of *Ube3a* would underlie a broad range of behavioral phenotypes in AS mice. In the present study, we instead found a larger role of *Ube3a* deletion from glutamatergic neurons in motor coordination, measured by rotarod and open field behavior, and innate species-specific behaviors such as marble burying. Furthermore, glutamatergic loss of UBE3A appears to mediate alterations in sleep patterning and induces some sleep fragmentation, while UBE3A loss from GABAergic neurons only caused fragmented sleep. Interestingly, glutamatergic reinstatement of *Ube3a* also rescued the decreased REM sleep observed in AS mice, as estimated by the PiezoSleep system. While this study identified some roles of GABAergic neurons in nest building behavior and sleep fragmentation, our data largely suggest a divergence of the neural circuitry underlying the motor, innate behavior, and sleep phenotypes of AS mice from the circuitry responsible for seizure susceptibility and cortical EEG patterns.

While our data suggest a large role of glutamatergic loss of UBE3A in behavioral phenotypes, they also highlight the challenges of studying cell type contributions to complex behaviors. For example, deletion of *Ube3a* from VGLUT2-expressing neurons caused decreased distance traveled in the open field test, but *Ube3a* reinstatement in this population provided no rescue of open field behavior. Conversely, *Ube3a* deletion from glutamatergic neurons caused no significant effect in nest building behavior and only subtle alterations in sleep patterning, but gene replacement in these neurons substantially improved performance in both measures. This discordance between *Ube3a* deletion and reinstatement in sleep and nest building behavior suggests that UBE3A loss from glutamatergic neurons is necessary for AS behavioral phenotypes but is not in itself sufficient to drive behavioral impairment. This could imply an important role for a population of neurons that was not assessed in this study, such as neuromodulatory populations. Indeed, serotoninergic, cholinergic, and hypocretin/orexin neurons are critical for establishing sleep–wake patterns and could also orchestrate presleep behaviors like nest building (Eban-Rothschild et al., 2018). While these nuanced results are difficult to fully interpret, they largely point toward a prominent role of glutamatergic neuron loss of UBE3A in many AS behavioral phenotypes.

One limitation of behavioral battery studies such as this is the multifaceted nature of the behavioral tests used. For example, marble burying and nest building are innate behavioral programs that require the coordinated activity of multiple circuit nodes, many of which are likely unknown, but also require sufficient motor ability. Therefore, it is difficult to attribute performance on a particular task to a specific behavioral domain. As glutamatergic loss of UBE3A caused deficits in multiple tasks that require intact gross motor function (open field, marble burying, and rotarod), we intuit that this neuronal population exerts a prominent role in motor performance.

This study also revealed key contributions of glutamatergic loss of UBE3A to sleep phenotypes. Here, we demonstrate that the PiezoSleep system can detect similar sleep phenotypes in AS model mice to those previously reported using EEG/EMG recordings, such as their characteristic lack of “siesta” and decreased REM sleep during the light cycle (Colas et al., 2005; Ehlen et al., 2015; Shi et al., 2022; Lee et al., 2023). Importantly, AS model mice also display dysfunctional sleep homeostasis, measured by their response to sleep deprivation (Ehlen et al., 2015) and 24 h light or dark exposure (Shi et al., 2022). These sleep behaviors were not assessed in the present study but should be addressed in future experiments. Additionally, it must be noted that the PiezoSleep system has only been validated for its accuracy of sleep staging in one publication, which was conducted using rats (Vanneau et al., 2021). Furthermore, this system has been validated for studying sleep using simultaneous EEG/EMG in only wild-type mice, raising the possibility that it may less accurately measure sleep in mouse models with motor impairments. While the piezoelectric system is not the gold standard measure of sleep–wake behavior, our ability to reproduce key AS sleep phenotypes with this system further supports its use as a high-throughput method to study sleep–wake behavior in genetic mouse models.

While much work has been done to dissect the neural circuitry mediating sleep–wake transitions and circadian rhythms, the mechanisms governing sleep–wake behavior during the active period are less understood. One recent study revealed a role for GABAergic vasoactive intestinal peptide (VIP)-expressing neurons in the hypothalamic suprachiasmatic nucleus (SCN) in shaping the “siesta” period sleep of mice during the dark cycle (Collins et al., 2020). However, in the present study, deletion of maternal *Ube3a* from GABAergic neurons, presumably including those of the SCN, tended to lengthen the “siesta” period rather than eliminate it. Additionally, neuronal silencing of the paternal *Ube3a* allele is partially relaxed in the SCN relative to other brain regions (Ehlen et al., 2015; Jones et al., 2016). These data suggest the lack of “siesta” in AS mice may be mediated by circuitry outside the SCN, such as those involved in the accumulation of sleep pressure.

This study focuses on the contributions of glutamatergic and GABAergic neurons to AS-related behaviors, but it is possible that certain phenotypes are influenced by altered activity of other neuronal populations, such as neuromodulatory cells. Indeed, AS model mice display altered dopamine release in the mesolimbic and nigrostriatal pathways (Riday et al., 2012), as well as altered firing properties and synaptic transmission in medium spiny neurons of the striatum, which receives substantial dopaminergic input (Hayrapetyan et al., 2014; Rotaru et al., 2023). Interestingly, the majority of midbrain dopaminergic neurons transiently express VGLUT2 during development and are targeted by the Vglut2-Cre mouse line (Steinkellner et al., 2018), indicating behavioral effects of UBE3A loss from midbrain dopaminergic neurons would be reflected by the Vglut2-Cre::*Ube3a^mFLOX/p+^* mouse model.

UBE3A is expressed in virtually all brain regions and neuron types and is paternally silenced in nearly all mature neurons (Judson et al., 2014; Gonzalez Ramirez et al., 2024), making the AS mouse model well suited to study the behavioral consequences of neuron type-specific manipulations. Our previous studies demonstrated a major role for GABAergic loss of UBE3A in seizure susceptibility and cortical oscillation patterns of AS model mice, suggesting these phenotypes are mediated by altered E–I balance broadly favoring excitation. In the present study, our data largely implicate glutamatergic loss of UBE3A in mediating many AS behavioral phenotypes, suggesting a different modality of E–I imbalance in the manifestation of these symptoms. Excitatory and inhibitory populations, however, are entangled and complex, and their contributions to behavior can be studied on various scales from inter-region connectivity to regional microcircuitry. These data on the broad cell types driving behaviors can serve as an entry point to more focused studies examining the key neuronal subtypes and brain regions involved. Taken alongside our previous findings, these data underscore the importance of *UBE3A* reinstatement strategies effectively targeting both excitatory and inhibitory neuron populations for optimal symptom improvement in AS individuals.

## Synthesis

Reviewing Editor: Fabienne Poulain, University of South Carolina Decisions are customarily a result of the Reviewing Editor and the peer reviewers coming together and discussing their recommendations until a consensus is reached. When revisions are invited, a fact-based synthesis statement explaining their decision and outlining what is needed to prepare a revision will be listed below. The following reviewer(s) agreed to reveal their identity: Caterina Montani. Note: If this manuscript was transferred from JNeurosci and a decision was made to accept the manuscript without peer review, a brief statement to this effect will instead be what is listed below.

Thank you for submitting your manuscript to eNeuro. After careful consideration, the reviewers and I both agree that your study investigating the effects of maternal deletion of Ube3A selectively in GABAergic or glutamatergic neurons in male and female mice is interesting and novel, with important implications for the understanding of Angelman's syndrome (AS) and neurodevelopmental disorders. However, reviewers also agree that the following points need to be addressed before your manuscript can be considered for publication.

**Major points:**

1) The **statistical testing of the data** needs to be revised. The same dataset seems to be tested in different ways without proper consideration that all tests are being done from the same collected data. For instance, ANOVA with Sidak multiple comparison post hoc tests were used for open field test (5-minute binned data), then One-way ANOVA with Tukey post hoc multiple comparisons tests were used for open field test (30-minute total) and subsequently open field data is tested again using t-tests when looking at 30 minutes total. The same is seen for marble burying (manual count vs threshold analysis) and rotarod.

1a) For Figures 1B, 1C, 2A and 2B, it does not seem necessary to test the same data in different ways to achieve the same important biological conclusion. Choosing one way to perform the analysis would avoid having to correct for multiple testing of the same data and be more appropriate. The analysis shown on the left is preferable as it shows the data fully.

1b) For rotarod testing in Figures 2C and 3C, testing the same data in different ways leads to differences in the conclusions regarding statistical significance. The authors need to elaborate as to why they think this is. There also seems to be a ceiling effect with several individual animals clustering at the top on the aggregated data on the right side.

1c) For analyses in which there are 3-different genotypes with multiple sampling over time (Figures 3,5, 7), the correct initial test is a repeated measures ANOVA with two main effects, with subsequent one-way repeated measures ANOVAs only if significant main effects or interactions are found. Subsequent t-tests can also be performed but may not be necessary to support the main findings. Any secondary testing needs to be corrected for multiple comparisons.

2) There are some concerns regarding **sleep phenotyping and data analysis**. Sleep phenotyping was performed using a non-contact device, the Piezo sleep. It is important for the authors to acknowledge that this is not the gold-standard for rodent sleep studies (which is polysomnography, PSG). Nonetheless this device is valid and valuable for screening for genotype effects and thus, the resulting data is interesting. Sleep staging (eg NREM vs REM) using this device, however, is not widely accepted in the field for two reasons: 1) there is only one available publication supporting the accuracy of PiezoSleep for sleep staging relative to PSG (Vanneau et al., 2021), which was done in rats and not mice, and 2) the PiezoSleep system was only validated in WT mice without motor deficits. Given the reliance of the system on movement, one must be careful with mice that present with a motor phenotype such as this one. In addition, this study did not evaluate sleep homeostasis (response to SD), which is a point of inconsistency between two previous studies (Shi et al., 2022, and Ehlen et al., 2015) in terms of the effect of maternal Ube3a deletion on sleep. Thus, the sleep phenotyping is incomplete. The authors need to acknowledge the limitations of the sleep study more fully.

2a) Why is Figure 5 the only one containing Gad2Cre only controls and why are those animals only on the top panels?

2b) The bout analysis is puzzling. Why were the bouts averaged in 4-hour bins? If sleep time analysis is done in one-hour bins, then bout duration analysis should also be done in 1-hour bins unless there is proper justification for not doing so.

2c) To facilitate the comparison between time sleeping and bout duration, the authors should include a plot of time sleeping expressed as total time rather than as a percentage, in the supplementary figures, at least for AS animals.

2d) The authors need to elaborate further on why maternal deletion in glutamatergic neurons does not produce a difference in sleep time (Figure 6), but the rescue in glutamatergic neurons does rescue the lack of siesta (Figure 7).

2e) The authors should provide stronger support for their interpretation of the sleep phenotype in Vglut2-Cre::Ube3amFLOX/p+ compared to Gad2-Cre::Ube3amFLOX/p+ mice, considering that 1) the comparison between Vglut2-Cre::Ube3amFLOX/p+ , Gad2-Cre::Ube3amFLOX/p+ mice and their respective controls (Vglut2-Cre [males] and Gad2-Cre) does not appear significantly different when looking at the plots, 2) the trends between the controls themselves appear somewhat different, particularly in the percentage of time spent sleeping, and 3) the rescue obtained through the glutamatergic reinstatement of Ube3a is partial.

2f) Do the AS mice in Figure 5 have the same genotype as the purple mice in Figure 7?

2g) The same issue regarding testing the same data different ways discussed in point 1 arises here as well. Sleep data is highly time of day dependent, thus we recommend removing panels B and D in Figures 4-9. Removing these panels will not change the overall conclusions of the study.

3) Special attention should be directed to the consistency between introduction, experimental questions, and results.

3a) The abstract refers to investigating a sex-specific effect, but no results are mentioned on this aspect in the abstract and not even in the final section of the introduction. The authors should decide whether to emphasize this point and, if so, address it more consistently.

3b) In the introduction and in the significant statement paragraph, the authors focus on excitatory-inhibitory (E/I) imbalance. However, it is not appropriate to dedicate so much attention to this topic, as the manuscript does not mechanistically explore the functional or circuit-level effects of the genetic manipulation performed. The authors state *"While deletion of Ube3a from GABAergic neurons, presumably by altering E-I balance, plays a key role in seizure and EEG phenotypes of AS model mice..."*. The authors should either reduce this part, move it to the discussion, or better support it with additional data demonstrating a clear link between their manipulation and neurodevelopmental E/I imbalance.

3c) Experimental questions should be more clearly defined in the abstract and introduction, similar to the clarity provided by the title and in the discussion with the statement: "This study focuses on the contributions of glutamatergic and GABAergic neurons to AS-related behaviors."

4) The authors should clarify their interpretation of Figure 2D (*"Lastly, Vglut2-Cre::Ube3amFLOX/p+ mice were tested in the nest building assay, in which they showed comparable performance to littermate controls, complementing the significant deficits observed in the Gad2-Cre::Ube3amFLOX/p+ mice"*) and of Figure 3D (*"Finally, when evaluated in the nest building task, Vglut2-Cre::Ube3amSTOP/p+ mice demonstrated a partial phenotypic rescue, complementing the qualitatively partial effect of Ube3a deletion from Gad2+ neurons (Fig. 1E) when compared to typical behavior of AS mice"*). It is not clear what *"complementing"* refers to. Data here seem to point not to an hypothesized complementarity between the behavioral effect mediated by glutamatergic and GABAergic systems, but rather to the involvement of other cell subtypes contributing to nest-building behavior, as these other subtypes are Ube3a- in AS mice but Ube3a+ in Vglut2-Cre::Ube3amFLOX/p+ mice. This last observation should also be considered as a limitation of the study.

5) The open field test also provides data on the time the tested mouse spends in the center of the arena, which serves as an indication of exploratory anxiety levels. The authors should also present these data if they collected them.

6) IHC analysis and quantifications showing the cell-specific absence and reinstatement of Ube3a in experimental animals should be included, unless previously published.

7) Given the number of genetic models used, the authors should provide a brief summary of the nomenclature in the material and methods section to facilitate the interpretation of the results, clearly specifying which genotype was used as control (e.g. Gad2-Cre or Ube3aFLOX/p+).

**Minor points:**

1) Full outputs of statistical analysis with exact p-values including post-hoc tests before and after multiple testing correction should be made available as a supplementary table. In some instances, it is hard to know exactly what the p-value was based on the figure legends or text and whether a multiple testing correction was applied appropriately or not.

2) Were all the sleep quantifications done using Piezoelectric? If so, it would be better to homogenize the figure legends. In some cases, the method is indicated, in others it is not.

3) All behavioral data should be deposited in public repositories. For data types in which a standard repository is not available, a server such as Zenodo can be used. Rodent sleep data should be made available through the National Sleep Research Resource (NSRR) which is an NIH approved standard repository for both human and rodent sleep data of all kinds.

4) Sidak corrections for multiple testing are usually very conservative. Benjamini-Hochberg could be used instead to better balance false positives with false negatives.

5) Please indicate which figure and panel the following part refers to: *"Notably, we observed significantly decreased marble burying in the Vglut2-Cre mice when compared to Cre-negative littermates [post hoc tests: Controls vs. Vglut2-Cre: left: t(30.32) = 3.185, p = 0.0100, right: t(30.99) = 3.335, p = 0.0066]. Nonetheless, Vglut2-Cre::Ube3amFLOX/p+ mice displayed significantly decreased marble burying when compared to Vglut2-Cre controls [post hoc tests: Vglut2-Cre vs. Vglut2-Cre::Ube3amFLOX/p+: left: t(12.04) = 3.302, p = 0.0182, right: t(13.28) = 4.219, p = 0.0029], suggesting a key role for Ube3a loss in glutamatergic neurons for this phenotype."*

## Author Response

We thank the reviewers for taking the time to carefully review this manuscript and for their detailed comments. We have made substantial revisions, summarized in blue below, that have improved the quality of the paper. Page and line number callouts refer to those in the marked up version of the revised manuscript.

Major points:

1) The statistical testing of the data needs to be revised. The same dataset seems to be tested in different ways without proper consideration that all tests are being done from the same collected data. For instance, ANOVA with Sidak multiple comparison post hoc tests were used for open field test (5-minute binned data), then One-way ANOVA with Tukey post hoc multiple comparisons tests were used for open field test (30-minute total) and subsequently open field data is tested again using t-tests when looking at 30 minutes total. The same is seen for marble burying (manual count vs threshold analysis) and rotarod.

We agree that we should only use one method of statistical analysis per experiment to test the major hypothesis for each dataset. To analyze our data in the most appropriate manner, we consulted a biostatistician from the North Carolina Translational and Clinical Sciences Institute (NC TraCS) and now follow their suggested changes. Accordingly, we have reworked our presentation of Figures 1, 2, and 3 to provide uniformity in the visualization and analyses of our data (see revised manuscript and below). We have included only the necessary statistical tests to compare relevant differences between groups. To aid readers in their interpretation of our data and for full data transparency, we have kept the data broken out by sex and genotype in the Extended Data. Notably, throughout the manuscript we now present statistical analyses only in the main figures and not the supplemental figures, with the exception of pointing out the lack of effect of the Gad2-Cre line versus controls in Supplemental Figure 1F when we explored possible effects using an additional cohort of mice.

1a) For Figures 1B, 1C, 2A and 2B, it does not seem necessary to test the same data in different ways to achieve the same important biological conclusion. Choosing one way to perform the analysis would avoid having to correct for multiple testing of the same data and be more appropriate. The analysis shown on the left is preferable as it shows the data fully.

We have kept the threshold analyses of marble burying behavior in our main figures, and we have removed the manual count analyses of these same data from the main figures. However, we have kept these additional analyses in the Extended Data to highlight the rigor of our approach and to demonstrate that our conclusions are reproducible across subjective and objective analyses, both of which are commonly used in the field to analyze marble burying.

For our rotarod analyses, we have removed the data collapsing the acquisition and retest trial days, as those data are already depicted by showing motor performance on the individual trials.

1b) For rotarod testing in Figures 2C and 3C, testing the same data in different ways leads to differences in the conclusions regarding statistical significance. The authors need to elaborate as to why they think this is. There also seems to be a ceiling effect with several individual animals clustering at the top on the aggregated data on the right side.

We feel that there is no discrepancy in the data: both the data from the 5-trial presentations and the data collapsed across the 5 presentations resulted in significant main effects of genotype. However, in one case, correcting for a higher number of post hoc comparisons reduced statistical power such that trials on individual days did not always reach statistical significance. Regardless, as requested, we show only one statistical analysis of the data represented across individual trials. This data presentation is the most transparent, and does not change our conclusions.

We agree that there is a ceiling effect in the rotarod task, particularly in the last 2 trials, because wild-type mice learn this task very well. This is almost certainly the case in many publications using the 3-30 rpm version of the task, but groups rarely report individual data points. We have chosen to report the individual data points in the extended data for maximum transparency.

1c) For analyses in which there are 3-different genotypes with multiple sampling over time (Figures 3,5, 7), the correct initial test is a repeated measures ANOVA with two main effects, with subsequent one-way repeated measures ANOVAs only if significant main effects or interactions are found. Subsequent t-tests can also be performed but may not be necessary to support the main findings. Any secondary testing needs to be corrected for multiple comparisons.

For Figure 3, we now use a two-way repeated measures ANOVA to analyze the open field, rotarod, and nest building data. As significant main effects or interactions were observed by ANOVA, we performed post hoc Tukey's multiple comparisons tests to assess for overall differences between genotypes. These post hoc comparisons are represented in each figure panel as brackets connecting the relevant genotypes. We agree that subsequent t-tests are not required for the interpretation of these data, so these have been removed. These data are now analyzed in a manner consistent with norms in the field (Sonzogni et al., 2020; Milazzo et al., 2021).

Our changes to Figures 5 and 7 are included with the responses to the sleep data (Major comment 2g, below).

2) There are some concerns regarding sleep phenotyping and data analysis. Sleep phenotyping was performed using a non-contact device, the Piezo sleep. It is important for the authors to acknowledge that this is not the gold-standard for rodent sleep studies (which is polysomnography, PSG). Nonetheless this device is valid and valuable for screening for genotype effects and thus, the resulting data is interesting. Sleep staging (eg NREM vs REM) using this device, however, is not widely accepted in the field for two reasons: 1) there is only one available publication supporting the accuracy of PiezoSleep for sleep staging relative to PSG (Vanneau et al., 2021), which was done in rats and not mice, and 2) the PiezoSleep system was only validated in WT mice without motor deficits. Given the reliance of the system on movement, one must be careful with mice that present with a motor phenotype such as this one. In addition, this study did not evaluate sleep homeostasis (response to SD), which is a point of inconsistency between two previous studies (Shi et al., 2022, and Ehlen et al., 2015) in terms of the effect of maternal Ube3a deletion on sleep. Thus, the sleep phenotyping is incomplete. The authors need to acknowledge the limitations of the sleep study more fully.

These are excellent points, and we have incorporated them into a more thorough discussion of the limitations of our study (Page 29, lines 641-648).

2a) Why is Figure 5 the only one containing Gad2Cre only controls and why are those animals only on the top panels? As previously stated in the first paragraph of our Results section, we designed these experiments with the intention of collapsing control groups, as others in the field have routinely done. However, in some circumstances we detected unexpected behavioral consequences of Cre expression (in the absence of a cross to a Cre-dependent line). We only separated control groups in the main figure when we observed a significant effect of our Cre driver lines, as we thought it was important to show these Cre driver line effects both for data transparency and to serve as a caution to others in the field that some Cre driver lines themselves can have modest phenotypes. Therefore, we show the Gad2-Cre control group in Figure 5A, in which there is different sleep patterning in this group, but not in Figure 5C, in which this group shows comparable sleep bout duration to WT mice (as demonstrated in the extended data). We chose this strategy to be as transparent as possible, while not distracting the reader with separated control groups that are not required for data interpretation.

2b) The bout analysis is puzzling. Why were the bouts averaged in 4-hour bins? If sleep time analysis is done in one-hour bins, then bout duration analysis should also be done in 1-hour bins unless there is proper justification for not doing so.

Sleep bout data collected using the piezo system are typically quantified using 12-hour averages across the light and dark cycles (Lord et al., 2022; Xu et al., 2023; Houle et al., 2024; Shannon et al., 2024). This is because 1) hourly bout duration is highly variable, and 2) with smaller bin durations, there is an increased chance that a given sleep bout will fall on the transition between bins, thus artificially shortening the measured sleep bout duration. We chose to report our data in 4-hour bins, as have others (Bedard et al., 2023), to explore whether there is a temporal aspect to this behavior across the light and dark cycles. Using this, we revealed a robust decrease in sleep bout duration of AS mice across the light cycle, but in the dark cycle, we only observed a notable decrease in bout duration toward the end of the dark cycle, corresponding to the timing of the "siesta".

2c) To facilitate the comparison between time sleeping and bout duration, the authors should include a plot of time sleeping expressed as total time rather than as a percentage, in the supplementary figures, at least for AS animals.

We have added total time sleeping data to Extended Data Figure 4-1.

2d) The authors need to elaborate further on why maternal deletion in glutamatergic neurons does not produce a difference in sleep time (Figure 6), but the rescue in glutamatergic neurons does rescue the lack of siesta (Figure 7).

We have now elaborated on the discordance between Ube3a deletion and reinstatement in glutamatergic neurons in the Discussion (Page 28, lines 616-623), and see our response to comment 2e below.

2e) The authors should provide stronger support for their interpretation of the sleep phenotype in Vglut2-Cre::Ube3amFLOX/p+ compared to Gad2-Cre::Ube3amFLOX/p+ mice, considering that 1) the comparison between Vglut2-Cre::Ube3amFLOX/p+ , Gad2-Cre::Ube3amFLOX/p+ mice and their respective controls (Vglut2-Cre [males] and Gad2-Cre) does not appear significantly different when looking at the plots, 2) the trends between the controls themselves appear somewhat different, particularly in the percentage of time spent sleeping, and 3) the rescue obtained through the glutamatergic reinstatement of Ube3a is partial.

2e.1) It is interesting that neither glutamatergic nor GABAergic deletion of Ube3a alone is sufficient to drive a lack of "siesta" comparable to that of AS model mice. However, glutamatergic reinstatement largely rescues this phenotype in AS mice. This suggests that glutamatergic deletion of Ube3a is necessary, but not alone sufficient, to induce the lack of "siesta". We have elaborated on this in the Discussion (Page 28).

2e.2) We agree there are some unusual trends between control groups when split by sex. While we are sufficiently powered to detect relevant differences between genotypic groups, we are underpowered to make sex-specific comparisons between control groups, which are not necessary for our primary scientific questions. We include these data mainly to demonstrate that our major findings are not driven by overt sex-specific effects. For clarity, we have now removed statistical testing from Extended Data Figures 4-1, 5-1, 6-1, and 8-1.

2e.3) We now clarify in the Results (Page 23, lines 517-518) that glutamatergic reinstatement of Ube3a largely, but incompletely, restores sleep patterning.

2f) Do the AS mice in Figure 5 have the same genotype as the purple mice in Figure 7? The AS mice in Figure 4 are the canonical Ube3am-/p+ mouse model. The mice of the same color in Figure 7 are Ube3amSTOP/p+ mice, in which a stop codon has been inserted within intron 3 of Ube3a. In the absence of Cre, these mice are equivalent to Ube3am-/p+ mice in terms of UBE3A expression. We have clarified this on Page 23, lines 508-510.

2g) The same issue regarding testing the same data different ways discussed in point 1 arises here as well. Sleep data is highly time of day dependent, thus we recommend removing panels B and D in Figures 4-9. Removing these panels will not change the overall conclusions of the study.

We agree that for behaviors such as rotarod and open field, comparing main effects is sufficient to draw the necessary conclusions. However, for sleep analysis, we have two primary questions to address with these data: 1) is there a difference in total amount of sleep in the light or dark cycle, and 2) is there a difference in the hourly pattern of sleep? Therefore, it is necessary to report both hourly sleep and 12-hour averages. Hourly and 12-hour sleep quantifications are both reported in numerous mouse studies of sleep behavior using the piezo sleep system (Holth et al., 2019; Lord et al., 2022; Bedard et al., 2023; Leu et al., 2024; Martin et al., 2024; McGowan et al., 2024), and in studies using EEG/EMG analyses of sleep behavior (Clasadonte et al., 2017; Anaclet et al., 2018; Holth et al., 2019; Fritz et al., 2021; Xu et al., 2023; Medina et al., 2024).

Demonstrating total sleep in the light and dark cycles is also required for proper interpretation of sleep bout duration, as decreased bout duration in the context of unchanged total sleep implies sleep fragmentation. This conclusion could not be reached without the data presented in Panel B of each sleep figure.

Our data show that AS model mice display a robust decrease in estimated REM sleep in the light cycle, while this effect is not apparent in the dark cycle. This phenotype has also been shown using EEG/EMG analyses of sleep (Colas et al., 2005; Lee et al., 2023). Since AS model mice only show decreased estimated REM sleep in the light cycle, analyses by only 24-hour main effect would not sufficiently answer our question of whether glutamatergic reinstatement of Ube3a rescues the decreased light cycle REM sleep of AS model mice. Therefore, the most appropriate way to statistically assess the day/night pattern of estimated REM sleep in this mouse model is to present averages of light and dark cycle sleep, and compare across groups with two-way repeated measures ANOVA and Sidak post hoc multiple comparisons tests.

3) Special attention should be directed to the consistency between introduction, experimental questions, and results.

3a) The abstract refers to investigating a sex-specific effect, but no results are mentioned on this aspect in the abstract and not even in the final section of the introduction. The authors should decide whether to emphasize this point and, if so, address it more consistently.

We included that both sexes were used in our study to be in accordance with the eNeuro guidelines, requiring that the abstract "provide a concise summary of the... methodology (including the species and sex studied)..." We have altered our phrasing to avoid suggesting that we are focused on sex-specific differences (Page 2, line 49).

3b) In the introduction and in the significant statement paragraph, the authors focus on excitatory-inhibitory (E/I) imbalance. However, it is not appropriate to dedicate so much attention to this topic, as the manuscript does not mechanistically explore the functional or circuit-level effects of the genetic manipulation performed. The authors state "While deletion of Ube3a from GABAergic neurons, presumably by altering E-I balance, plays a key role in seizure and EEG phenotypes of AS model mice...". The authors should either reduce this part, move it to the discussion, or better support it with additional data demonstrating a clear link between their manipulation and neurodevelopmental E/I imbalance.

This is a great point. We have significantly reduced the amount of text discussing excitatory-inhibitory imbalance, as we are not directly studying this in the paper, and we agree that speculation on this topic is better suited for the Discussion. However, since we are studying the roles of excitatory and inhibitory neuron deletion of Ube3a, we do believe some description of the E-I imbalance hypothesis in the introduction is helpful in contextualizing the study.

3c) Experimental questions should be more clearly defined in the abstract and introduction, similar to the clarity provided by the title and in the discussion with the statement: "This study focuses on the contributions of glutamatergic and GABAergic neurons to AS-related behaviors." We have more clearly stated the primary questions in the Abstract (Page 2, line 49) and Introduction (Page 5, lines 109-111).

4) The authors should clarify their interpretation of Figure 2D ("Lastly, Vglut2-Cre::Ube3amFLOX/p+ mice were tested in the nest building assay, in which they showed comparable performance to littermate controls, complementing the significant deficits observed in the Gad2-Cre::Ube3amFLOX/p+ mice") and of Figure 3D ("Finally, when evaluated in the nest building task, Vglut2-Cre::Ube3amSTOP/p+ mice demonstrated a partial phenotypic rescue, complementing the qualitatively partial effect of Ube3a deletion from Gad2+ neurons (Fig. 1E) when compared to typical behavior of AS mice"). It is not clear what "complementing" refers to. Data here seem to point not to an hypothesized complementarity between the behavioral effect mediated by glutamatergic and GABAergic systems, but rather to the involvement of other cell subtypes contributing to nest-building behavior, as these other subtypes are Ube3a- in AS mice but Ube3a+ in Vglut2-Cre::Ube3amFLOX/p+ mice. This last observation should also be considered as a limitation of the study.

We can see why this sentence in the text would be unclear, so we have removed it. The purpose of the statement was to point out that Vglut2-Cre::Ube3amFLOX/p+ mice showed deficits in each motor behavior except for nest building, which was the only behavior impaired in Gad2-Cre::Ube3amFLOX/p+ mice. Therefore, these two models largely complement one another.

5) The open field test also provides data on the time the tested mouse spends in the center of the arena, which serves as an indication of exploratory anxiety levels. The authors should also present these data if they collected them.

We extracted center time from our open field videos, and have included these data in Extended Data 1-1, 2-1, and 3-2. We did not observe a significant effect of center time in Gad2-Cre::Ube3amFLOX/p+ mice, Vglut2-Cre::Ube3amFLOX/p+ mice, or Ube3amSTOP/p+ mice. In line with this, a recent meta-analysis of 238 mice showed no significant difference in center time between AS and WT mice (Tanas et al., 2022).

Additionally, we believe differences in center time should be interpreted with caution in mouse models with motor impairment, such as this one, as differences in center time could be driven by locomotor dysfunction rather than anxiety-like behavior (Stanford, 2007).

6) IHC analysis and quantifications showing the cell-specific absence and reinstatement of Ube3a in experimental animals should be included, unless previously published.

This has been added to Figures 1, 2, and Extended Data 3-1.

7) Given the number of genetic models used, the authors should provide a brief summary of the nomenclature in the material and methods section to facilitate the interpretation of the results, clearly specifying which genotype was used as control (e.g. Gad2-Cre or Ube3aFLOX/p+).

We have included a table of the experimental manipulation, terminology used, and sample sizes for each genotypic group for each figure as Table 1.

Minor points:

1) Full outputs of statistical analysis with exact p-values including post-hoc tests before and after multiple testing correction should be made available as a supplementary table. In some instances, it is hard to know exactly what the p-value was based on the figure legends or text and whether a multiple testing correction was applied appropriately or not.

We now include a summary of all statistical tests and results organized by figure number as Extended Data Figure 1-2.

2) Were all the sleep quantifications done using Piezoelectric? If so, it would be better to homogenize the figure legends. In some cases, the method is indicated, in others it is not.

We have now added "piezoelectric" to each sleep figure legend.

3) All behavioral data should be deposited in public repositories. For data types in which a standard repository is not available, a server such as Zenodo can be used. Rodent sleep data should be made available through the National Sleep Research Resource (NSRR) which is an NIH approved standard repository for both human and rodent sleep data of all kinds.

We will deposit our data into the Carolina Digital Repository for open access after this manuscript has been accepted.

4) Sidak corrections for multiple testing are usually very conservative. Benjamini-Hochberg could be used instead to better balance false positives with false negatives.

This is a great suggestion, however we are comfortable using the more conservative statistical test. For the comparisons most important to our conclusions, including comparisons of 1-hour time bins in sleep testing, Sidak is sufficient to detect the most robust differences between groups.

5) Please indicate which figure and panel the following part refers to: "Notably, we observed significantly decreased marble burying in the Vglut2-Cre mice when compared to Cre-negative littermates [post hoc tests: Controls vs. Vglut2-Cre: left: t(30.32) = 3.185, p = 0.0100, right: t(30.99) = 3.335, p = 0.0066]. Nonetheless, Vglut2-Cre::Ube3amFLOX/p+ mice displayed significantly decreased marble burying when compared to Vglut2-Cre controls [post hoc tests: Vglut2-Cre vs. Vglut2-Cre::Ube3amFLOX/p+: left: t(12.04) = 3.302, p = 0.0182, right: t(13.28) = 4.219, p = 0.0029], suggesting a key role for Ube3a loss in glutamatergic neurons for this phenotype." We thank the reviewers for catching this. This text has been changed since we have altered the marble burying data presentation (see reviewer comment 1a). We now reference Figure 2D (pages 16-17).

References Anaclet C, De Luca R, Venner A, Malyshevskaya O, Lazarus M, Arrigoni E, Fuller PM (2018) Genetic Activation, Inactivation, and Deletion Reveal a Limited And Nuanced Role for Somatostatin-Containing Basal Forebrain Neurons in Behavioral State Control. J Neurosci 38:5168-5181.

Bedard ML, Lord JS, Perez PJ, Bravo IM, Teklezghi AT, Tarantino LM, Diering GH, McElligott ZA (2023) Probing different paradigms of morphine withdrawal on sleep behavior in male and female C57BL/6J mice. Behav Brain Res 448:114441.

Clasadonte J, Scemes E, Wang Z, Boison D, Haydon PG (2017) Connexin 43-Mediated Astroglial Metabolic Networks Contribute to the Regulation of the Sleep-Wake Cycle. Neuron 95:1365-1380.e5.

Colas D, Wagstaff J, Fort P, Salvert D, Sarda N (2005) Sleep disturbances in Ube3a maternal-deficient mice modeling Angelman syndrome. Neurobiol Dis 20:471-478.

Fritz EM, Kreuzer M, Altunkaya A, Singewald N, Fenzl T (2021) Altered sleep behavior in a genetic mouse model of impaired fear extinction. Sci Rep 11:8978.

Holth JK, Fritschi SK, Wang C, Pedersen NP, Cirrito JR, Mahan TE, Finn MB, Manis M, Geerling JC, Fuller PM, Lucey BP, Holtzman DM (2019) The sleep-wake cycle regulates brain interstitial fluid tau in mice and CSF tau in humans. Science 363:880-884.

Houle S, Tapp Z, Dobres S, Ahsan S, Reyes Y, Cotter C, Mitsch J, Zimomra Z, Peng J, Rowe RK, Lifshitz J, Sheridan J, Godbout J, Kokiko-Cochran ON (2024) Sleep fragmentation after traumatic brain injury impairs behavior and conveys long-lasting impacts on neuroinflammation. Brain Behav Immun Health 38:100797.

Lee D, Chen W, Kaku HN, Zhuo X, Chao ES, Soriano A, Kuncheria A, Flores S, Kim JH, Rivera A, Rigo F, Jafar-Nejad P, Beaudet AL, Caudill MS, Xue M (2023) Antisense oligonucleotide therapy rescues disturbed brain rhythms and sleep in juvenile and adult mouse models of Angelman syndrome. eLife 12.

Leu C-L, Lam DD, Salminen AV, Wefers B, Becker L, Garrett L, Rozman J, Wurst W, Hrabě de Angelis M, Hölter SM, Winkelmann J, Williams RH (2024) A patient-enriched MEIS1 coding variant causes a restless legs syndrome-like phenotype in mice. Sleep 47.

Lord JS, Gay SM, Harper KM, Nikolova VD, Smith KM, Moy SS, Diering GH (2022) Early life sleep disruption potentiates lasting sex-specific changes in behavior in genetically vulnerable Shank3 heterozygous autism model mice. Mol Autism 13:35.

Martin SC, Joyce KK, Lord JS, Harper KM, Nikolova VD, Cohen TJ, Moy SS, Diering GH (2024) Sleep Disruption Precedes Forebrain Synaptic Tau Burden and Contributes to Cognitive Decline in a Sex-Dependent Manner in the P301S Tau Transgenic Mouse Model. eNeuro 11.

McGowan JC, Ladner LR, Shubeck CX, Tapia J, LaGamma CT, Anqueira-González A, DeFrancesco A, Chen BK, Hunsberger HC, Sydnor EJ, Logan RW, Yu T-S, Kernie SG, Denny CA (2024) Traumatic Brain Injury-Induced Fear Generalization in Mice Involves Hippocampal Memory Trace Dysfunction and Is Alleviated by (R,S)-Ketamine. Biol Psychiatry 95:15-26.

Medina E, Rempe MJ, Muheim C, Schoch H, Singletary K, Ford K, Peixoto L (2024) Sex differences in sleep deficits in mice with an autism-linked Shank3 mutation. Biol Sex Differ 15:85.

Milazzo C, Mientjes EJ, Wallaard I, Rasmussen SV, Erichsen KD, Kakunuri T, van der Sman ASE, Kremer T, Miller MT, Hoener MC, Elgersma Y (2021) Antisense oligonucleotide treatment rescues UBE3A expression and multiple phenotypes of an Angelman syndrome mouse model. JCI Insight 6.

Shannon T, Cotter C, Fitzgerald J, Houle S, Levine N, Shen Y, Rajjoub N, Dobres S, Iyer S, Xenakis J, Lynch R, de Villena FP-M, Kokiko-Cochran O, Gu B (2024) Genetic diversity drives extreme responses to traumatic brain injury and post-traumatic epilepsy. Exp Neurol 374:114677.

Sonzogni M, Zhai P, Mientjes EJ, van Woerden GM, Elgersma Y (2020) Assessing the requirements of prenatal UBE3A expression for rescue of behavioral phenotypes in a mouse model for Angelman syndrome. Mol Autism 11:70.

Stanford SC (2007) The Open Field Test: reinventing the wheel. J Psychopharmacol (Oxford) 21:134-135.

Tanas JK, Kerr DD, Wang L, Rai A, Wallaard I, Elgersma Y, Sidorov MS (2022) Multidimensional analysis of behavior predicts genotype with high accuracy in a mouse model of Angelman syndrome. Transl Psychiatry 12:426.

Xu M, Wang L, Wang Y, Deng J, Wang X, Wang F, Pan S, Zhao Y, Liao A, Wang X, Chen D, Shen J, Yang F, Li Y, Wang S (2023) Melatonin ameliorates sleep-wake disturbances and autism-like behaviors in the Ctnnd2 knock out mouse model of autism spectrum disorders. Genes Brain Behav 22:e12852.
