# Supplementary figures and images for: Cell Type-Specific Contributions of UBE3A to Angelman Syndrome Behavioral Phenotypes
Source: eNeuro. 2025 Sep 25;12(9):ENEURO.0453-24.2025. doi: 10.1523/ENEURO.0453-24.2025 (PMC12479163; doi:10.1523/ENEURO.0453-24.2025)

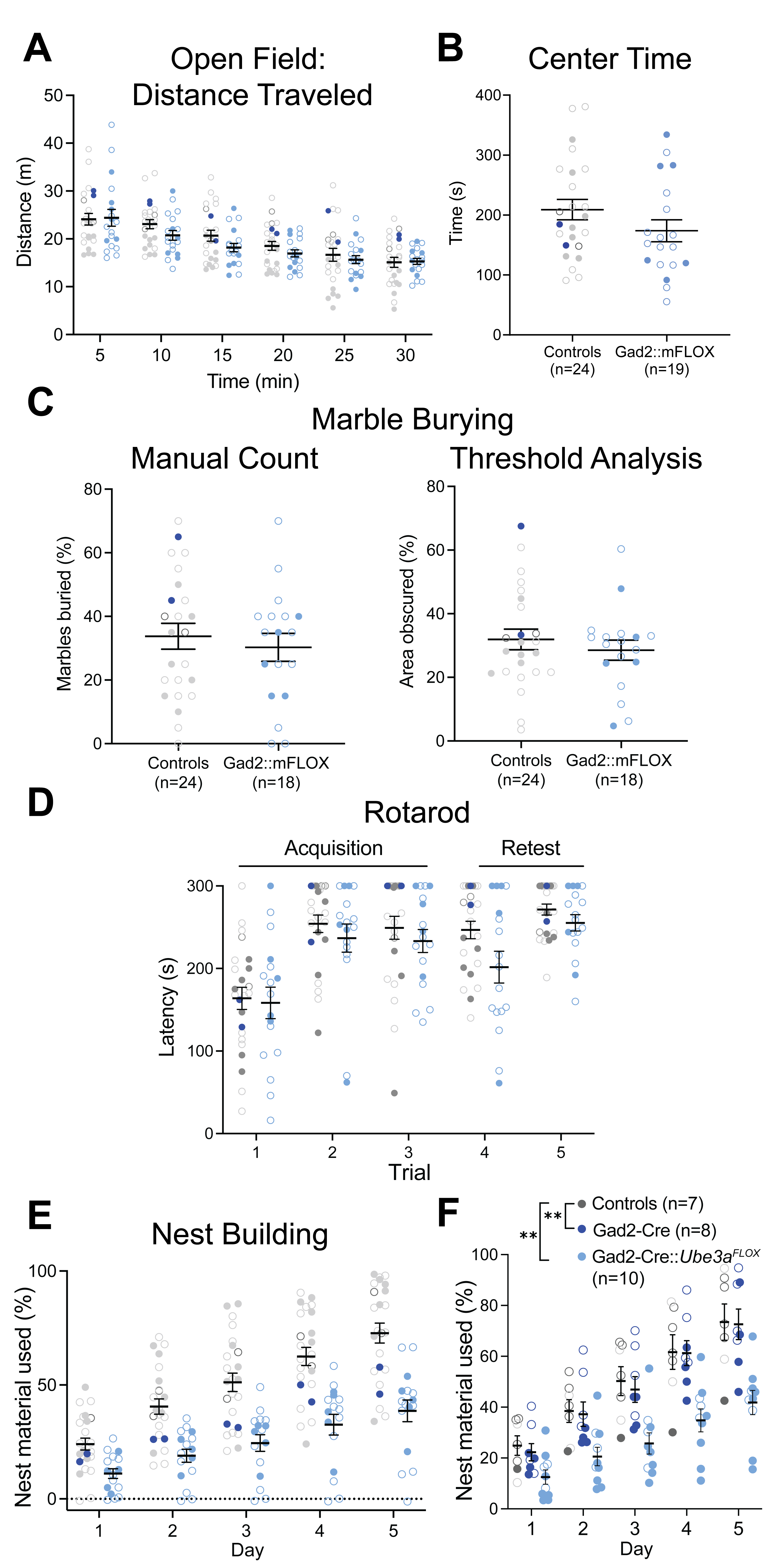

Supplement: Figure 1-1 — Gad2-Cre::Ube3amFLOX/p + ­ behavioral battery labeled by genotype and sex. Open circles = males, closed circles = females. Dark gray = WT, light gray = Ube3amFLOX/p+, dark blue = Gad2-Cre, light blue = Gad2-Cre::Ube3amFLOX/p + . (A) Distance traveled in the open field across 5-minute bins. (B) Total time in the center of the open field. (C) Quantification of marble burying behavior by manual count of buried marbles (left panel) and threshold-based analysis (right panel). (D) Latency to fall or first passive rotation on the rotarod across each acquisition (day 1) and retest (day 2) trial. (E) Quantification of percent nesting material used across 5-day test. (F) Nest building behavior from only maternal heterozygous Ube3am+/pFLOX litters with additional cohort added. WT and Ube3amFLOX/p+ controls (n = 7), Gad2-Cre (n = 8), Gad2-Cre::Ube3amFLOX/p+ (n = 10). Two-way RM ANOVA with Tukey’s post hoc comparisons for effect of genotype. Data presented as means ± SEM. *P < 0.05, **P < 0.01. Download Figure 1-1, TIF file. [file eneuro-12-ENEURO.0453-24.2025-s001.tif]

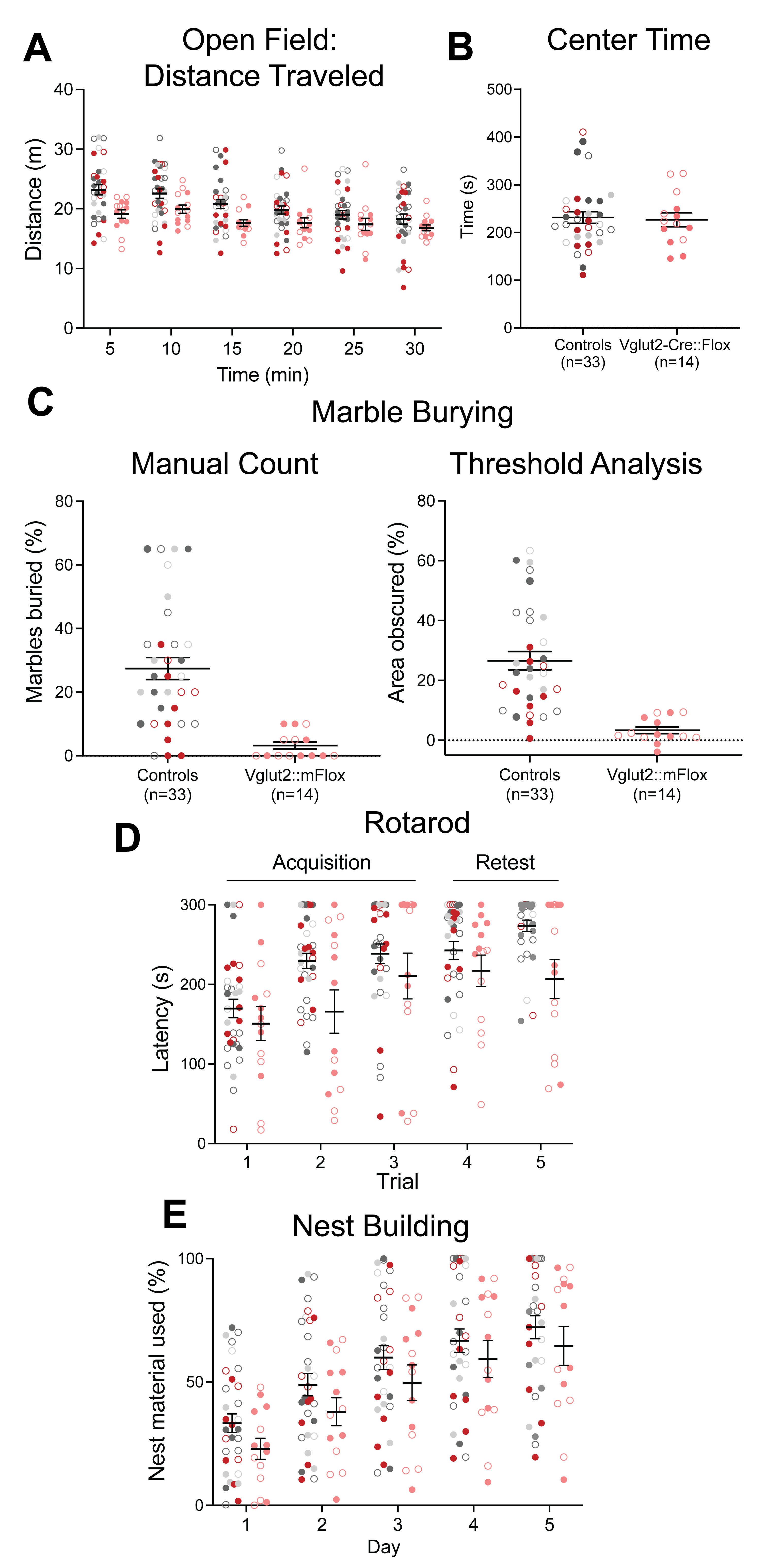

Supplement: Figure 2-1 — Vglut2-Cre::Ube3amFLOX/p + ­ behavioral battery labeled by genotype and sex. Open circles = males, closed circles = females. Dark gray = WT, light gray = Ube3amFLOX/p+, dark red = Vglut2-Cre, light red = Vglut2-Cre::Ube3amFLOX/p + . (A) Distance traveled in the open field across 5-minute bins. (B) Total time in the center of the open field. (C) Quantification of marble burying behavior by manual count of buried marbles (left panel) and threshold-based analysis (right panel). (D) Latency to fall or first passive rotation on the rotarod across each acquisition (day 1) and retest (day 2) trial. (E) Quantification of percent nesting material used across 5-day test. Download Figure 2-1, TIF file. [file eneuro-12-ENEURO.0453-24.2025-s003.tif]

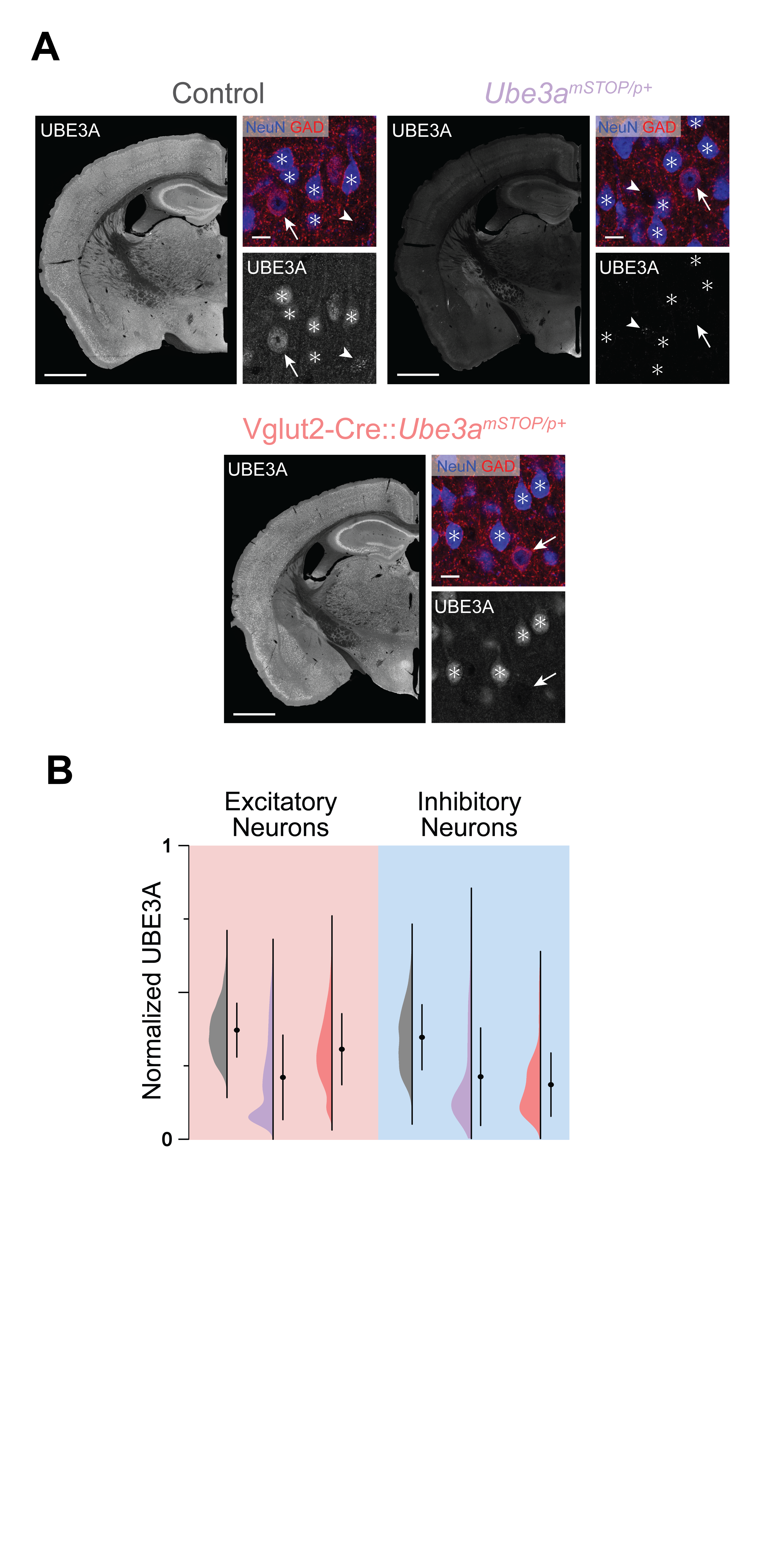

Supplement: Figure 3-1 — Glutamatergic neuron Ube3a reinstatement in Vglut2-Cre::Ube3amSTOP/p+ mice. (A) UBE3A immunostaining in Control, Ube3amSTOP/p+ and Vglut2-Cre::Ube3amSTOP/p+ mice. Asterisks indicate excitatory (NeuN+, GAD-) cells. Zoom images demonstrate cell type-specific UBE3A expression in the somatosensory cortex. Arrows indicate inhibitory (NeuN+, GAD+) neurons. Arrowheads indicate non-neuronal (NeuN-) cells with persistent, weak UBE3A expression in Ube3amSTOP/p+ mice. Hemi-section scale bar = 1 mm. Zoom image scale bar = 10 µm. (B) Nuclear UBE3A intensity of individual excitatory (NeuN+, GAD-) and inhibitory (NeuN+, GAD+) cells in the somatosensory cortex displayed as violin plots with means ± SD. Download Figure 3-1, TIF file. [file eneuro-12-ENEURO.0453-24.2025-s004.tif]

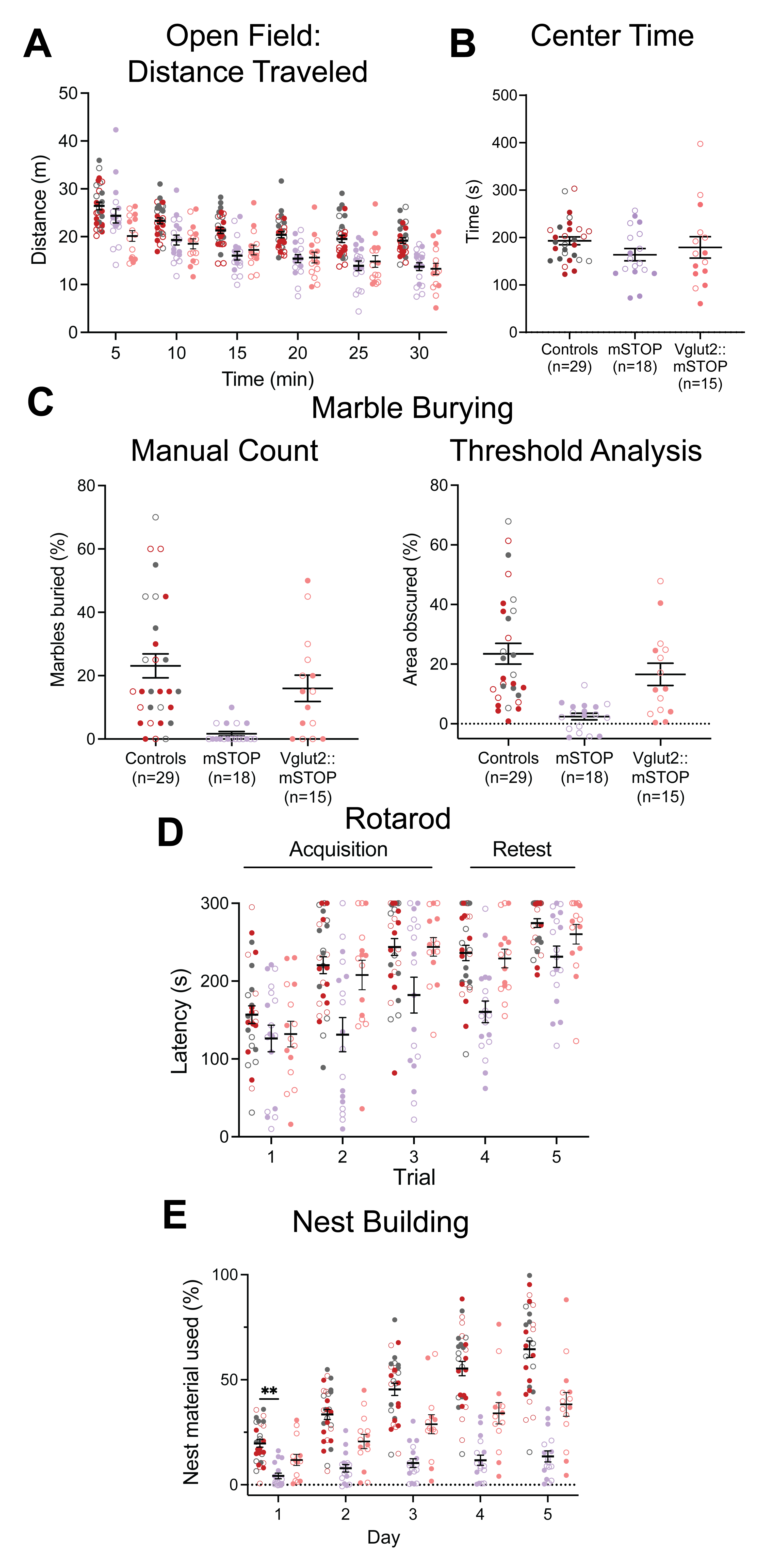

Supplement: Figure 3-2 — Vglut2-Cre::Ube3amSTOP/p+ behavioral battery labeled by genotype and sex. Open circles = males, closed circles = females. Gray = WT, dark red = Vglut2-Cre, purple = Ube3amSTOP/p+, light red = Vglut2-Cre::Ube3amSTOP/p + . (A) Distance traveled in the open field across 5-minute bins. (B) Total time in the center of the open field. (C) Quantification of marble burying behavior by manual count of buried marbles (left panel) and threshold-based analysis (right panel). (D) Latency to fall or first passive rotation on the rotarod across each acquisition (day 1) and retest (day 2) trial. (E) Quantification of percent nesting material used across 5-day test. Download Figure 3-2, TIF file. [file eneuro-12-ENEURO.0453-24.2025-s005.tif]

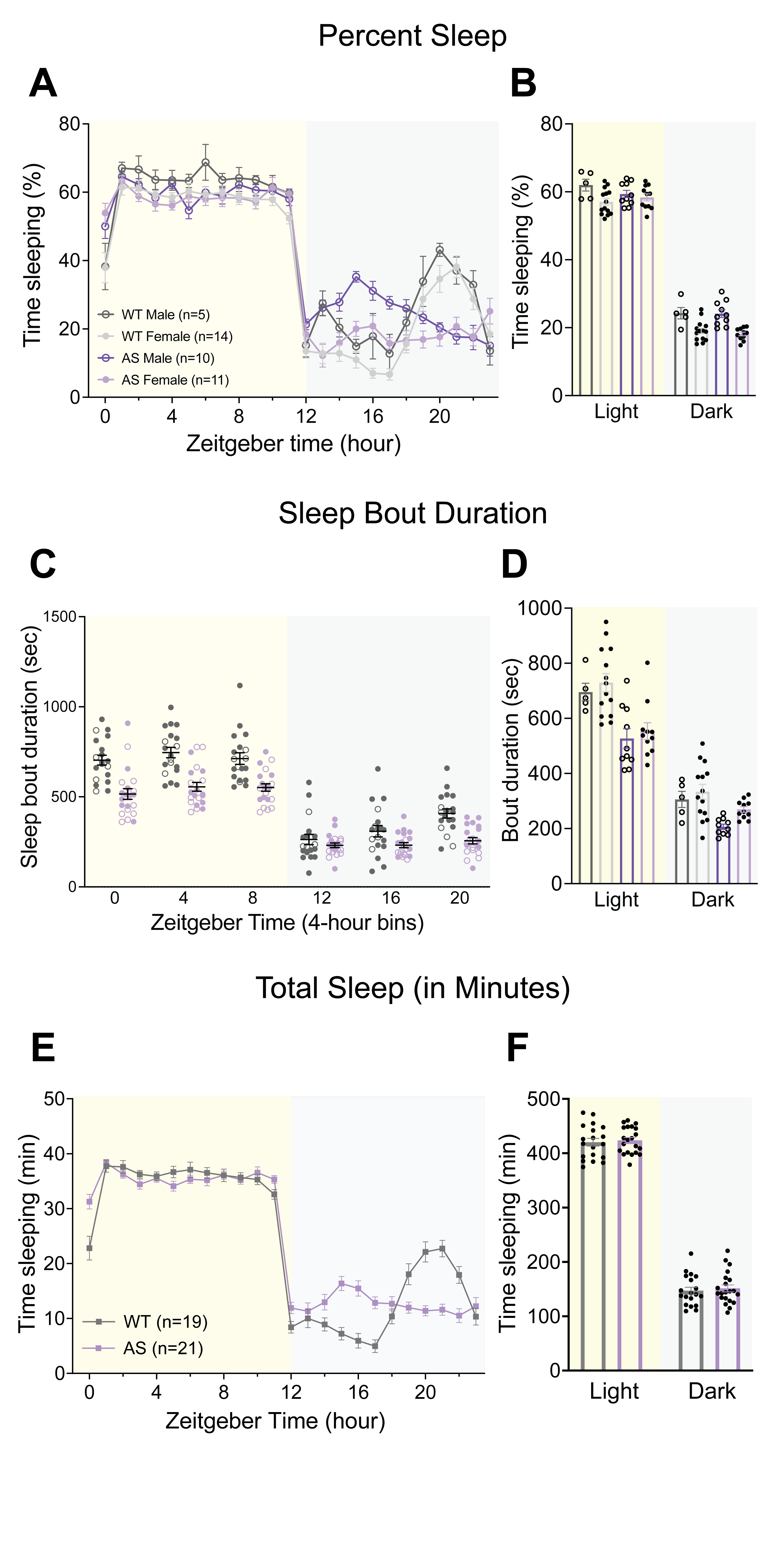

Supplement: Figure 4-1 — AS mouse sleep behavior separated by sex. Open circles = males, closed circles = females. (A) Piezoelectric quantification of hourly percent sleep in male and female AS and WT mice. (B) Average percent sleep across light and dark cycles. (C) Mean sleep bout durations across 4-hour time bins. (D) Mean sleep bout duration during light and dark cycle, averaged from 4-hour bins. (E) Piezoelectric quantification of hourly percent sleep presented in minutes. (F) Average percent sleep across light and dark cycles presented in minutes. Data presented as means ± SEM. Download Figure 4-1, TIF file. [file eneuro-12-ENEURO.0453-24.2025-s006.tif]

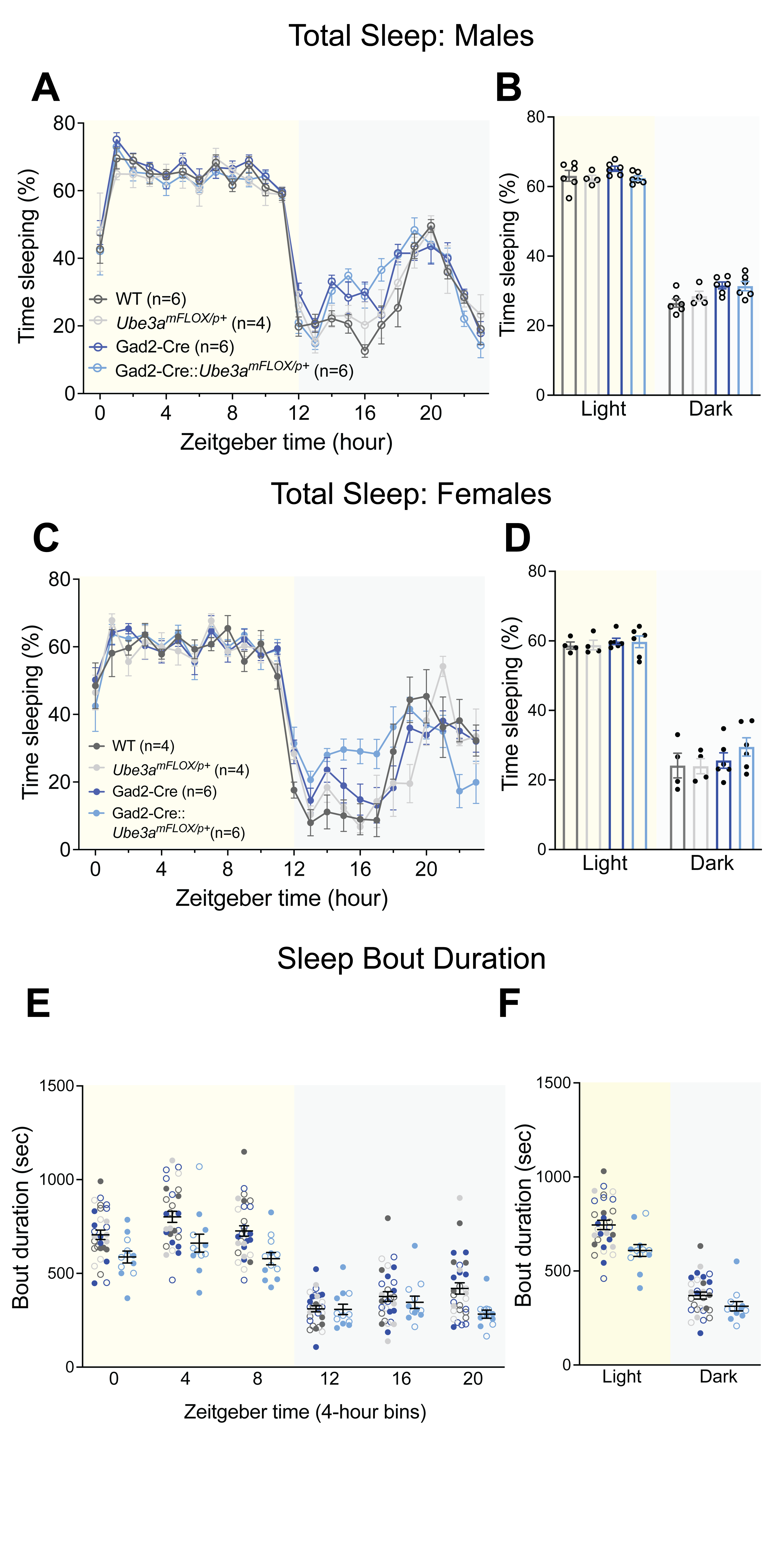

Supplement: Figure 5-1 — Gad2-Cre::Ube3amFLOX/p + ­ sleep behavior separated by genotype and sex. Open circles = males, closed circles = females. Dark gray = WT, light gray = Ube3amFLOX/p+, dark blue = Gad2-Cre, light blue = Gad2-Cre::Ube3amFLOX/p + . (A) Piezoelectric quantification of hourly percent sleep in males. (B) Average percent sleep across light and dark cycles in males. (C) Hourly percent sleep in females. (D) Average percent sleep across light and dark cycles in females. (E) Mean sleep bout durations across 4-hour time bins. (F) Mean sleep bout duration during light and dark cycle, averaged from 4-hour bins. Data presented as means ± SEM. Download Figure 5-1, TIF file. [file eneuro-12-ENEURO.0453-24.2025-s007.tif]

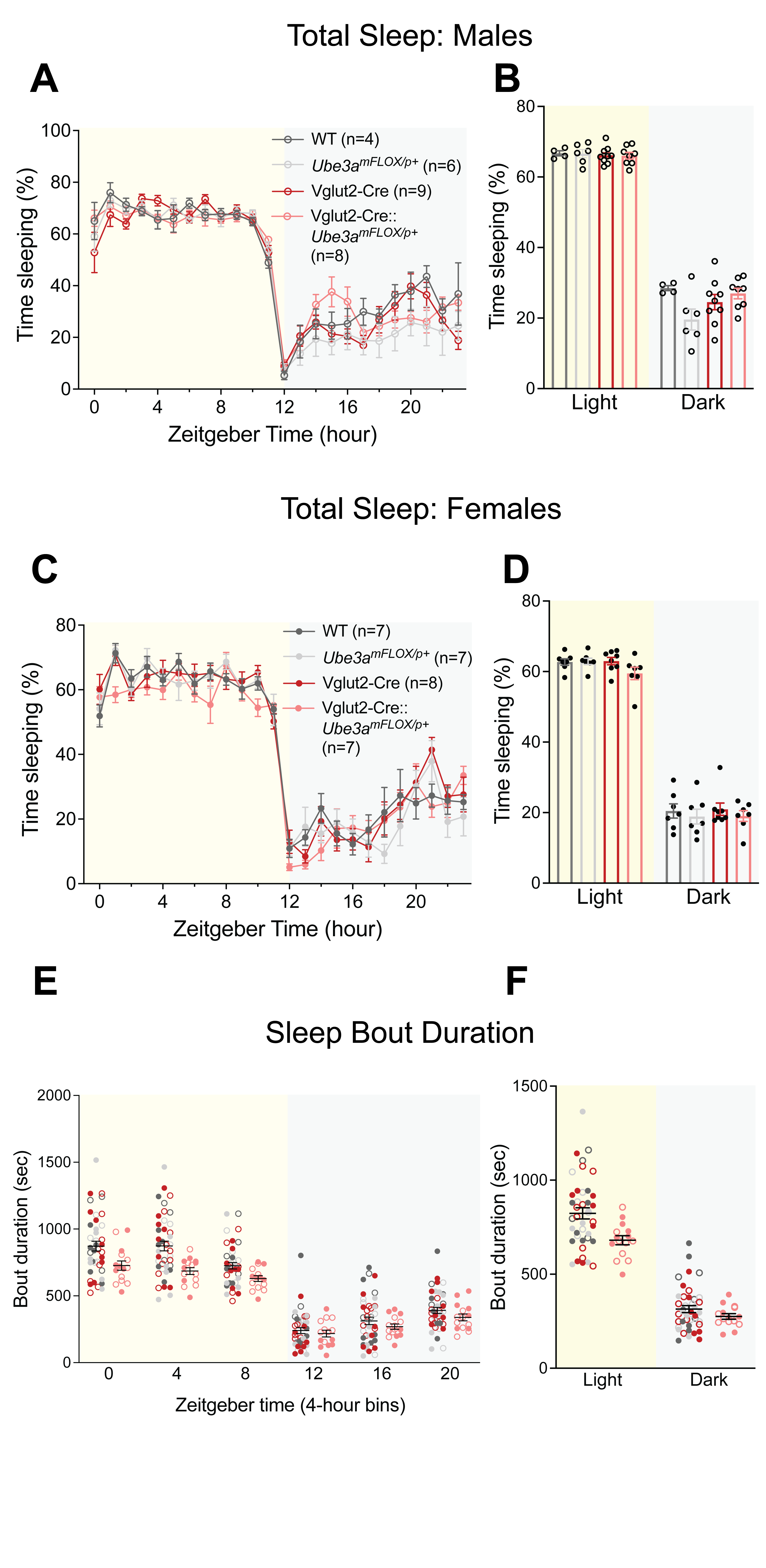

Supplement: Figure 6-1 — Vglut2-Cre::Ube3amFLOX/p + ­ sleep behavior separated by genotype and sex. Open circles = males, closed circles = females. Dark gray = WT, light gray = Ube3amFLOX/p+, dark red = Vglut2-Cre, light red = Vglut2-Cre::Ube3amFLOX/p + . (A) Piezoelectric quantification of hourly percent sleep in males. (B) Average percent sleep across light and dark cycles in males. (C) Hourly percent sleep in females. (D) Average percent sleep across light and dark cycles in females. (E) Mean sleep bout durations across 4-hour time bins. (F) Mean sleep bout duration during light and dark cycle, averaged from 4-hour bins. Data presented as means ± SEM. Download Figure 6-1, TIF file. [file eneuro-12-ENEURO.0453-24.2025-s008.tif]

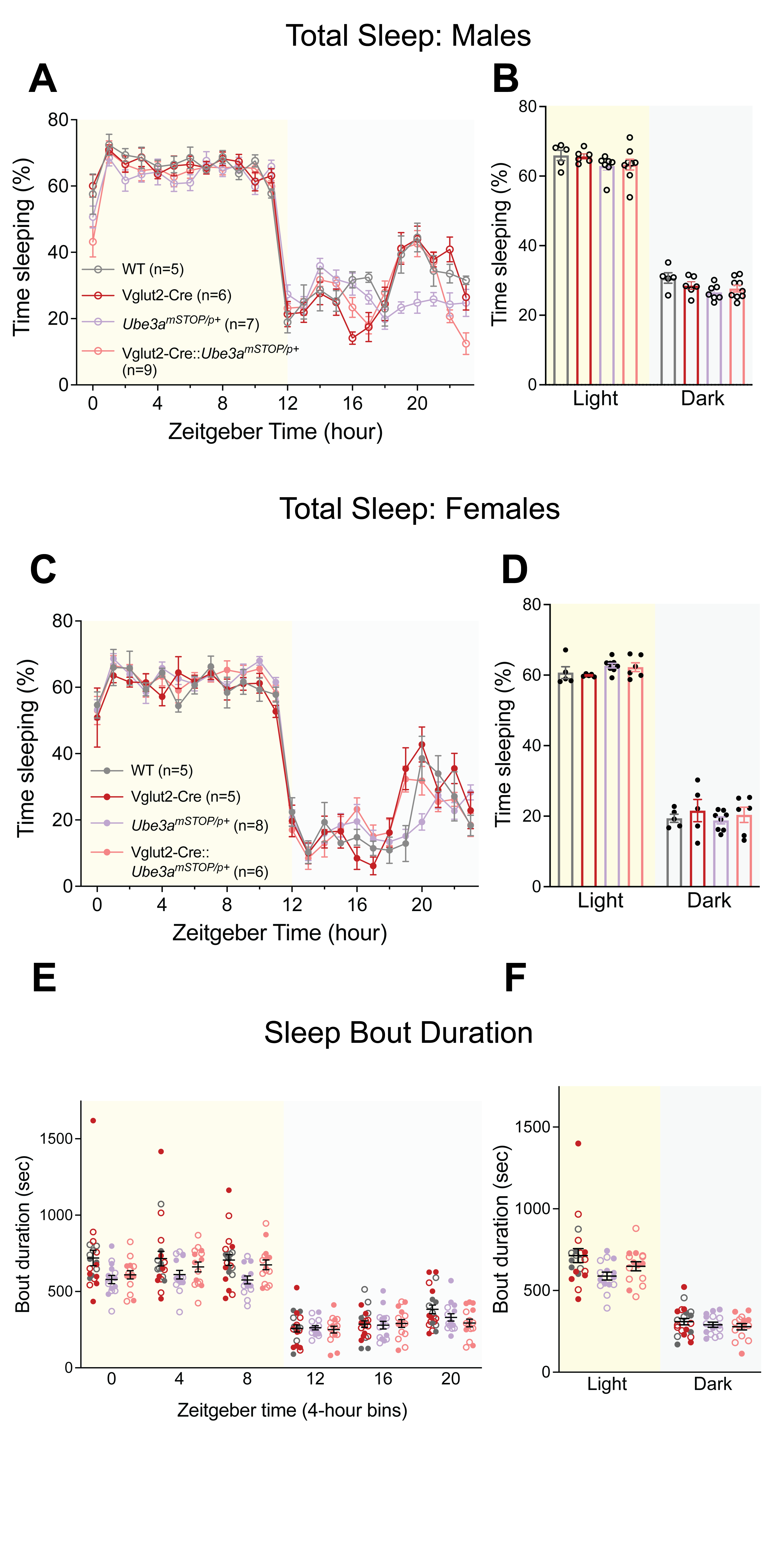

Supplement: Figure 7-1 — Vglut2-Cre::Ube3amSTOP/p + ­ sleep behavior separated by genotype and sex. Open circles = males, closed circles = females. Gray = WT, dark red = Vglut2-Cre, purple = Ube3amSTOP/p+, light red = Vglut2-Cre::Ube3amSTOP/p + . (A) Piezoelectric quantification of hourly percent sleep in males. (B) Average percent sleep across light and dark cycles. (C) Hourly percent sleep in females. (D) Average percent sleep across light and dark cycles in females. (E) Mean sleep bout durations across 4-hour time bins. (F) Mean sleep bout duration during light and dark cycle, averaged from 4-hour bins. Data presented as means ± SEM. Download Figure 7-1, TIF file. [file eneuro-12-ENEURO.0453-24.2025-s009.tif]

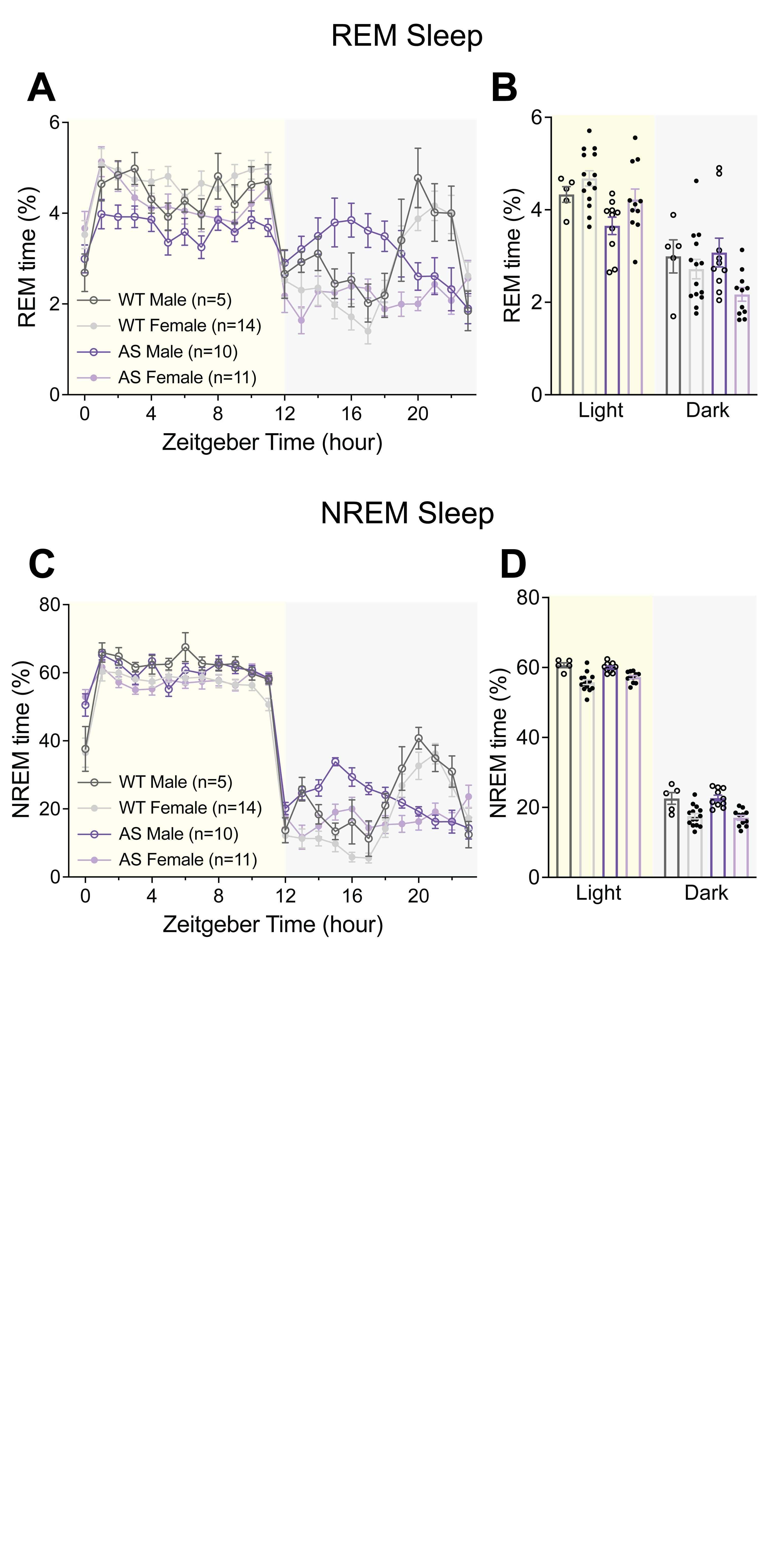

Supplement: Figure 8-1 — AS mouse estimated REM and NREM sleep separated by sex. Open circles = males, closed circles = females. (A) Piezoelectric estimation of hourly percent REM sleep in male and female AS and WT mice. (B) Average percent REM across light and dark cycles. (C) Estimated hourly percent NREM sleep in AS and WT mice. (D) Average percent NREM across light and dark cycles. Data presented as means ± SEM. Download Figure 8-1, TIF file. [file eneuro-12-ENEURO.0453-24.2025-s010.tif]

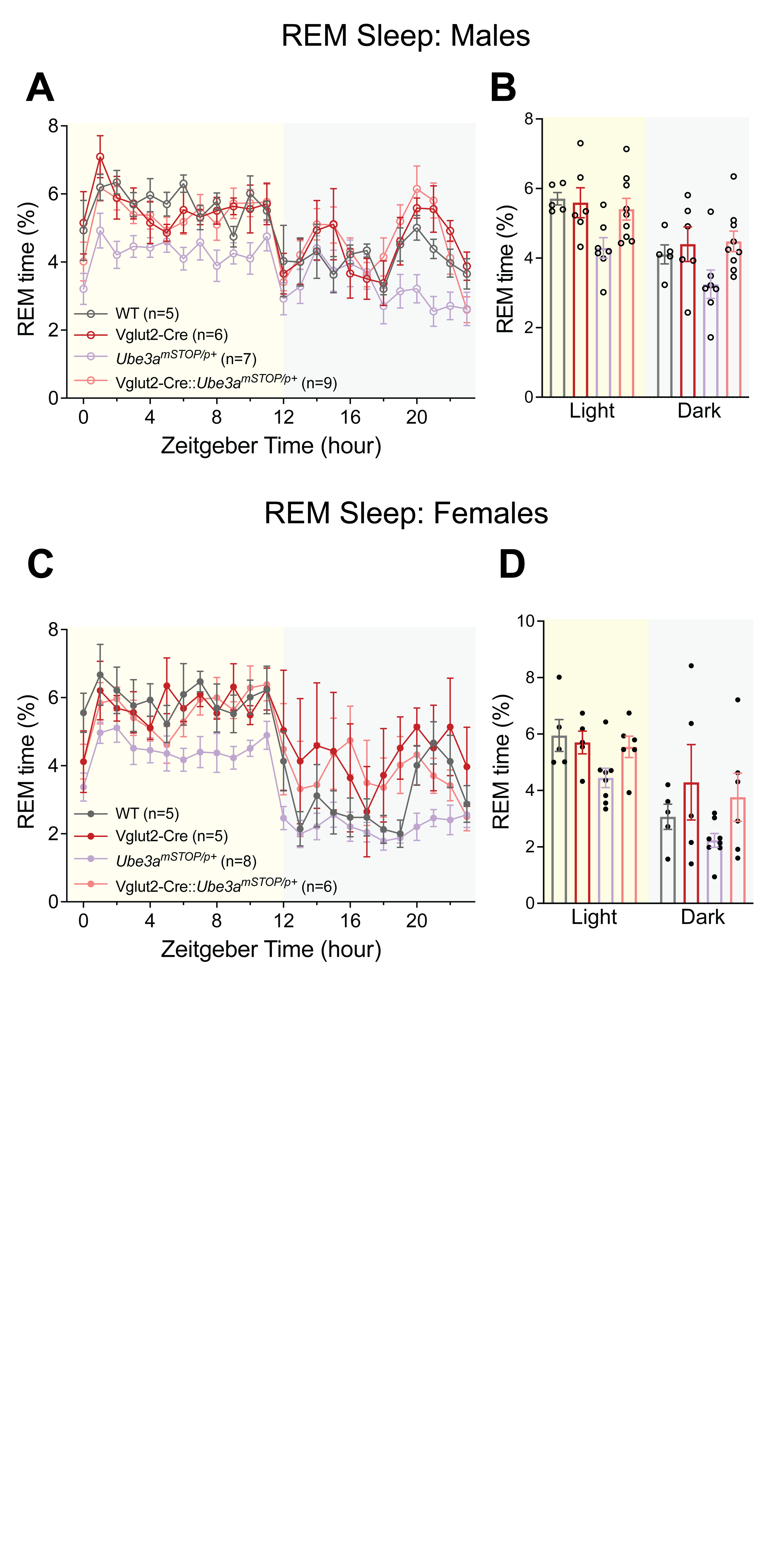

Supplement: Figure 9-1 — Vglut2-Cre::Ube3amSTOP/p + ­ REM sleep estimates separated by genotype and sex. (A) Piezoelectric estimation of hourly percent REM sleep in males. (B) Average percent REM across light and dark cycles in males. (C) Estimated hourly REM sleep in females. (D) Average percent REM across light and dark cycles in females. Data presented as means ± SEM. Download Figure 9-1, TIF file. [file eneuro-12-ENEURO.0453-24.2025-s011.tif]

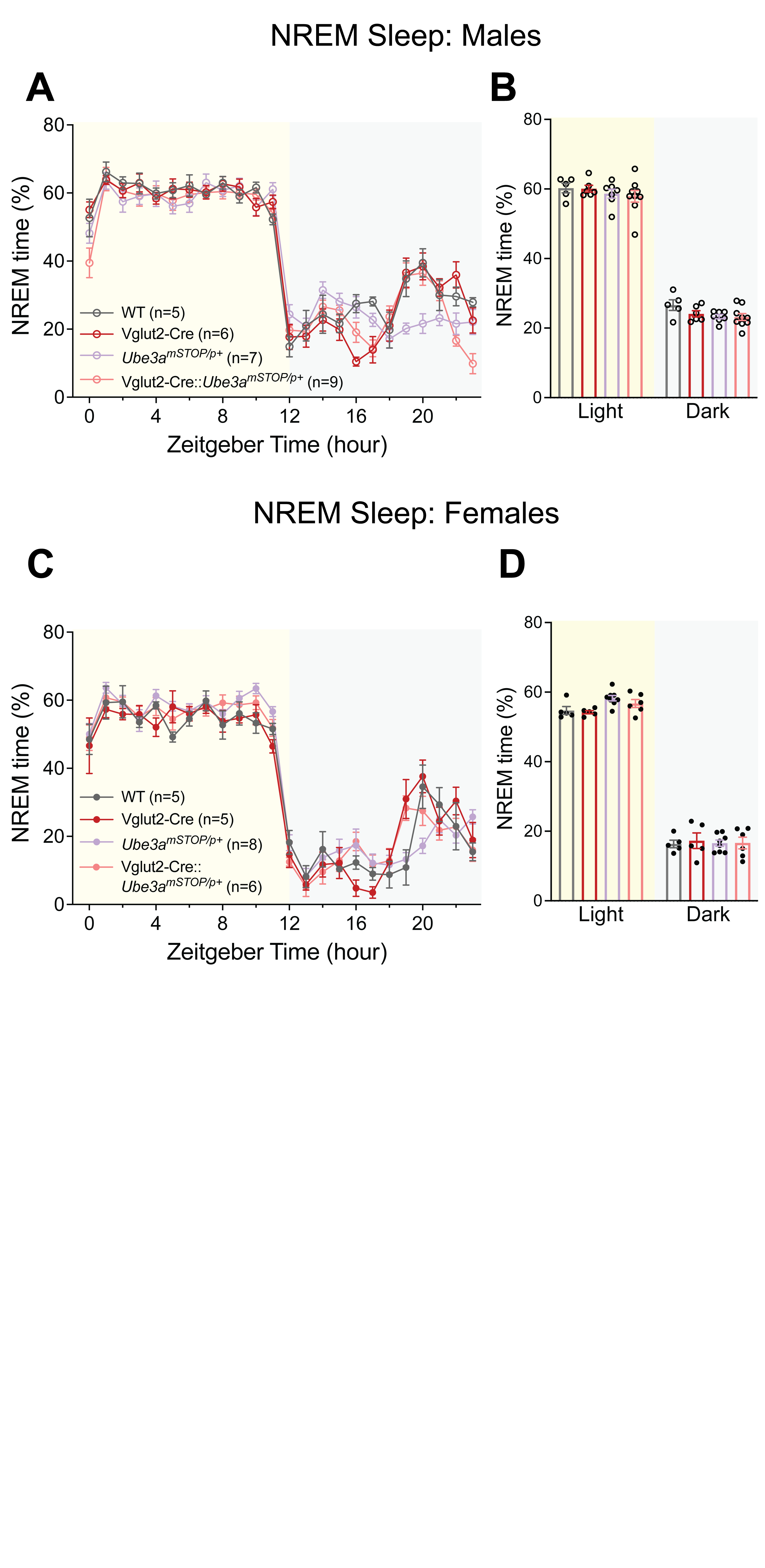

Supplement: Figure 9-2 — Vglut2-Cre::Ube3amSTOP/p + ­ NREM sleep estimates separated by genotype and sex. (A) Piezoelectric estimation of hourly percent NREM sleep in males. (B) Average percent NREM across light and dark cycles in males. (C) Estimated hourly NREM sleep in females. (D) Average percent NREM across light and dark cycles in females. Data presented as means ± SEM. Download Figure 9-2, TIF file. [file eneuro-12-ENEURO.0453-24.2025-s012.tif]
